# Hyper-brain hyper-frequency network topology dynamics when playing guitar in quartet

**DOI:** 10.3389/fnhum.2024.1416667

**Published:** 2024-06-11

**Authors:** Viktor Müller, Ulman Lindenberger

**Affiliations:** ^1^Center for Lifespan Psychology, Max Planck Institute for Human Development, Berlin, Germany; ^2^Max Planck UCL Centre for Computational Psychiatry and Ageing Research, Berlin, Germany; ^3^Max Planck UCL Centre for Computational Psychiatry and Ageing Research, London, United Kingdom

**Keywords:** within-and cross-frequency coupling, graph-theoretical approach, EEG hyperscanning, phase synchronization, hyper-brain hyper-frequency networks, social interaction

## Abstract

Ensemble music performance is a highly coordinated form of social behavior requiring not only precise motor actions but also synchronization of different neural processes both within and between the brains of ensemble players. In previous analyses, which were restricted to within-frequency coupling (WFC), we showed that different frequencies participate in intra- and inter-brain coordination, exhibiting distinct network topology dynamics that underlie coordinated actions and interactions. However, many of the couplings both within and between brains are likely to operate across frequencies. Hence, to obtain a more complete picture of hyper-brain interaction when musicians play the guitar in a quartet, cross-frequency coupling (CFC) has to be considered as well. Furthermore, WFC and CFC can be used to construct hyper-brain hyper-frequency networks (HB-HFNs) integrating all the information flows between different oscillation frequencies, providing important details about ensemble interaction in terms of network topology dynamics (NTD). Here, we reanalyzed EEG (electroencephalogram) data obtained from four guitarists playing together in quartet to explore changes in HB-HFN topology dynamics and their relation to acoustic signals of the music. Our findings demonstrate that low-frequency oscillations (e.g., delta, theta, and alpha) play an integrative or pacemaker role in such complex networks and that HFN topology dynamics are specifically related to the guitar quartet playing dynamics assessed by sound properties. Simulations by link removal showed that the HB-HFN is relatively robust against loss of connections, especially when the strongest connections are preserved and when the loss of connections only affects the brain of one guitarist. We conclude that HB-HFNs capture neural mechanisms that support interpersonally coordinated action and behavioral synchrony.

## Introduction

Music possesses an extraordinary ability to transcend boundaries, uniting people and creating harmonious connections. In the realm of music, quartet playing represents a sublime fusion of individual creativity and collective synergy. The magic unfolds when four skilled musicians, specifically guitarists, blend their unique styles and emotions, crafting melodies or sounds that resonate deeply with the human soul. Recent research indicates that synchronized brain activity, especially inter- or hyper-brain synchronization, accompanies coordinated behavior and plays a crucial role in social or musical interaction (Lindenberger et al., 2009; Sänger et al., 2012, 2013; Müller et al., 2013, 2018b; Keller et al., 2014; Müller and Lindenberger, 2019, 2022, 2023; Gugnowska et al., 2022). This synchrony can occur at the same or at different frequencies and can be indicated by within- and cross-frequency coupling (WFC and CFC, respectively). Such coupling or synchronization (i.e., within and between brains and within and between frequencies), reflecting the common integrated state of interactors and supporting hyper-brain hyper-frequency network (HB-HFN) activity, is of paramount importance. Moreover, these different types of oscillatory and network interactions reveal a superior degree of complexity that is essential for superorganismic organization and functioning (cf. Delius and Müller, 2023). The dynamics of the HB-HFN topology have a profound impact on the way we interact and respond to each other. However, the neural mechanisms responsible for facilitating coordinated actions between individuals and promoting social interaction remain elusive, especially when such interactions involve groups of more than two individuals, such as a guitar quartet or similar (Thompson and Varela, 2001; Frith and Frith, 2007; Hari and Kujala, 2009; Müller et al., 2018b, 2021; Müller, 2022).

The performance of even a simple piece of music demands precise control of timing to adhere to a hierarchical rhythmic structure. Additionally, musicians must skilfully control pitch to produce specific musical intervals based on frequency ratios. Music thus imposes unique demands on the nervous system, and an understanding of these demands can, in turn, provide insights into certain aspects of neural function (Zatorre et al., 2007). Furthermore, the expectations associated with rhythm and beat are an important component of temporal predictions in music. The ability to tune to an external auditory stimulus or a complex rhythm allows multiple individuals to synchronize their behavior in time by integrating the flow of information across different sensory modalities (Keller, 2008; Repp and Keller, 2008; Merker et al., 2009; Battich et al., 2020). All this necessitates an interaction of different frequencies and their integration in the entire network.

From our daily experiences, it is evident that social endeavors like music-making require learning and practice to become proficient and seamless. Through the process of learning, achieved by engaging in these social activities repeatedly, extraneous elements in interpersonal interactions are gradually refined, leading to enhanced fluidity in movement and improved motor skills. As highlighted by Müller (2022), there is an intrinsic relation between oscillatory activity, neural cell assemblies, and behavioral or cognitive entities. In the context of this relation, a hyper-brain cell assembly hypothesis has been suggested that states that cell assemblies can be formed not only within but also between brains, following roughly the same ‘Hebbian’ rules as within brains. Such hyper-brain cell assemblies, connecting two or more brains and triggering the simultaneous activation (firing) of neural components within these brains or the shared hyper-brain cell assembly, represent superordinate systems that encompass and integrate oscillatory activity within and between brains. This collective hyper-brain unit, which can also have a multidimensional or multilayer dynamic organization based on WFC and CFC within and between brains, serves as the foundation for social and interactive behaviors (Müller, 2022). Comparable concepts have also been discussed previously (Shamay-Tsoory, 2022). In this work, the author introduces the notion of *interbrain plasticity* or learning through interaction, where “interbrain plasticity” serves as a metaphor symbolizing the ability of inter-brain networks (which also rely on intra-brain connections) to reconfigure their functional organization in response to learning facilitated by interaction. Interbrain plasticity refers to the ability of multiple brains to adapt to experiences, resulting in both short- and long-term changes in inter-brain connectivity. These connectivity or coupling changes can then influence the behavioral repertoire of the individuals involved in the interaction (see also Mayo and Shamay-Tsoory, 2024). For example, think about a sports team or ensemble practicing together. As they train and play together over time, their brains become more synchronized in coordinating movements and strategies. This enhanced inter-brain connectivity reflects the plasticity of their collective neural networks, enabling them to perform better as a team or ensemble.

**Figure 1:**
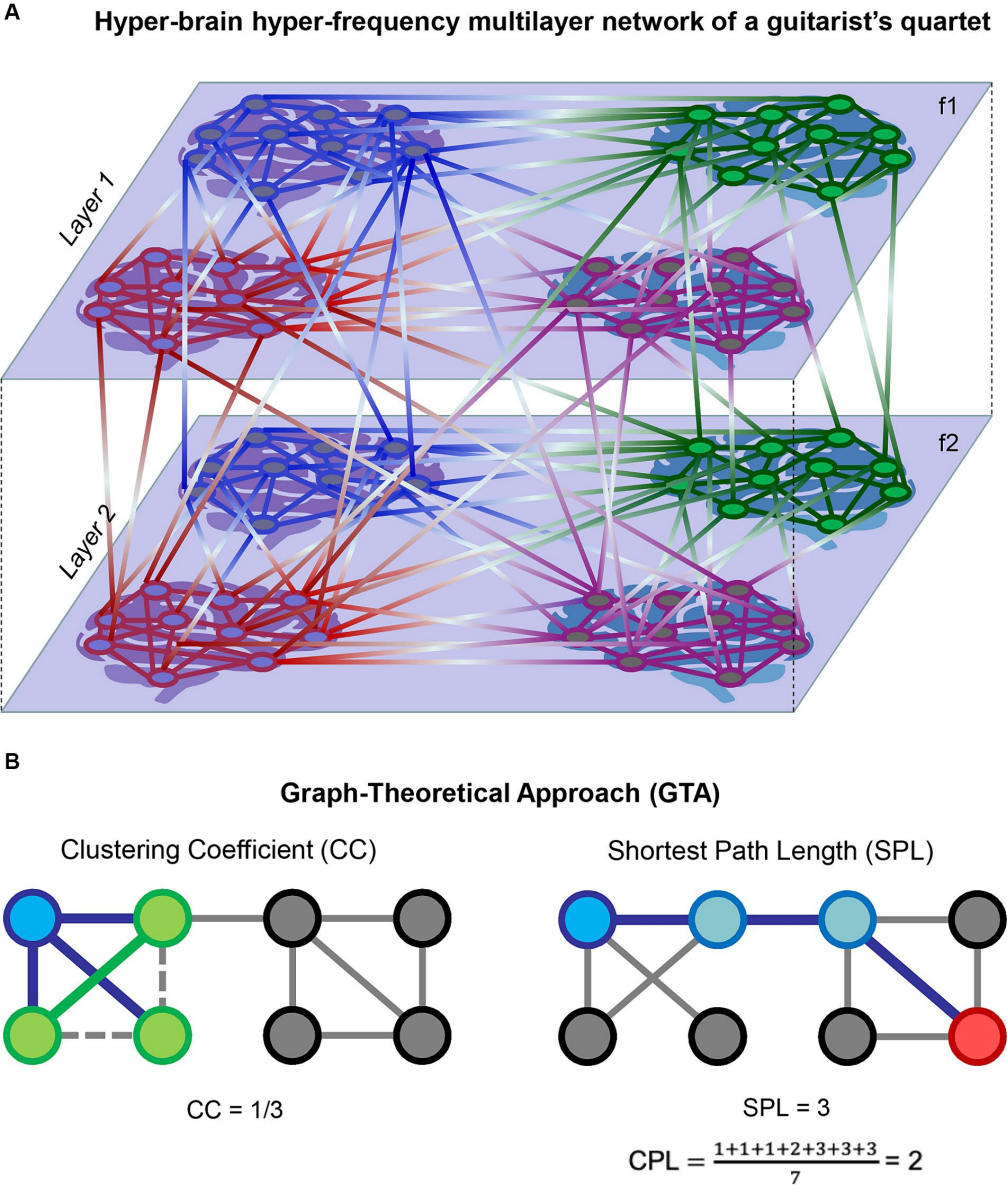
Schematic representation of HB-HFN and GTA. **(A)** Exemplary representation of a hyper-brain hyper-frequency multilayer network of a guitarist quartet. Four brains of the guitarist quartet within two layers with within- and between-layer connections are presented. The layers represent two different oscillation frequencies (f1 and f2), and connections within the layers indicate WFC, while connections between the layers indicate CFC. **(B)** Exemplary representation of two GTA measures: *CC* and *CPL*. On the left, *CC* for a target node (blue) is calculated as the ratio of one closed triangle to the three possible triangles, equaling 0.333. The three neighbors of the target node are presented in green. On the right, a shortest path length (*SPL*) is presented between the target node (blue) and another node (red) in the network, equaling 3. *CPL* is then calculated as the average of *SPL*s from the target node to all other nodes in the network, equaling 2. Note that for simplicity, a binary unweighted network was used in this representation. In the case of a directed weighted network, such as HB-HFN in our study, the direction and weights of links will play a role.

Complex networks (e.g., HFNs or HB-HFNs) can be regarded as multiplex or multilayer networks that have a specific multidimensional or multilayer network organization (De Domenico et al., 2013, 2015, 2016; Boccaletti et al., 2014; Kivelä et al., 2014; De Domenico, 2017; Pilosof et al., 2017; de Arruda et al., 2018). In this context, WFC represents communication within layers and CFC depicts communication between different layers (Brookes et al., 2016; Tewarie et al., 2016, 2021; De Domenico, 2017; Buldú and Porter, 2018; O’Neill et al., 2018; Tenney et al., 2021; Müller, 2022). Figure 1A exemplarily shows a complex two-layer four-brain network or HB-HFN of a guitar quartet. In a number of studies, it has been shown that multilayer networks can be represented as a supra-adjacency matrix, allowing conventional graph-theoretical approach (GTA) tools to be used to investigate their properties (Kivelä et al., 2014; Müller and Lindenberger, 2014; Brookes et al., 2016; De Domenico et al., 2016; Müller et al., 2016, 2019b; De Domenico, 2017; Müller, 2022). Figure 1B schematically illustrates two GTA measures, the clustering coefficient (*CC*) and characteristic path length (*CPL*), which we used in this work for network topology representation. In a concert study involving a quartet of guitarists and four audience members, the network topology dynamics (NTD) of the entire HB-HFN (quartet and audience) and the dynamical structure of guitar sounds showed specific guitar–guitar, brain–brain, and guitar–brain directional associations, indicating multilevel dynamics with upward and downward causation (Müller and Lindenberger, 2023).

The next important issue of interacting networks is their robustness, signifying the ability to maintain integrity and functionality of the network even after the removal of nodes or edges. This ability of the network is a prominent feature of most biological systems and social groups (Barabási and Pósfai, 2016; Liu et al., 2020; Bellingeri et al., 2020a), and may be useful for understanding interpersonal action coordination and the underlying hyper-brain networks. It has been reported that removing nodes according to weighted rank and also removing links in accordance with their weights produce the highest damage in real-world complex networks (Bellingeri and Cassi, 2018; Bellingeri et al., 2019, 2020a). Moreover, it has also been found that the robustness of the real-world complex networks against link removal is negatively correlated with link-weight heterogeneity (i.e., when the weights were randomly assigned to the links) and that the removal of a small fraction of strong links can rapidly decrease the efficiency and total flow in these networks (Bellingeri et al., 2019). It has also been reported that the removal of a single node or link has only limited impact on a network’s integrity, while the removal of several nodes or links can break a network into several isolated components or destroy the components of the network so that the network communication between remote nodes can no longer take place (Newman, 2003; Barabási and Pósfai, 2016). Most networks are robust against random vertex removal but considerably less robust to targeted removal of the highest-degree vertices (Newman, 2003).

In our previous analyses of data from a guitar quartet, which was restricted to WFC, we showed that different frequencies participate in intra- and interbrain coordination and exhibit different network topology dynamics that underlie coordinated actions and interactions (Müller et al., 2018b). However, many of the couplings both within and between brains are likely to operate across frequencies (Müller and Lindenberger, 2014; Müller et al., 2021; Müller, 2022). Hence, to obtain a more complete picture of hyper-brain interaction when guitarists play as a quartet, we considered both WFC and CFC that are used to construct a multilayer HB-HFNs integrating all the information flows within and between different frequencies oscillating at distinct cortical regions/brains and providing important details about ensemble interaction in terms of network topology dynamics (cf. Müller, 2022). Our hypothesis was that hyperbrain coupling strengths among the four guitarist brains would decrease with higher oscillation frequencies, thereby eliciting corresponding effects on the NTD. Furthermore, it has been shown that there is a specific coupling between musicians’ brains and musical instruments (Müller and Lindenberger, 2019, 2022, 2023). In this context, it is to be expected that guitar sounds not only correlate with, or predict each other, but that this correlation or prediction also concerns guitar–brain relations. To substantiate these relations (i.e., guitar–guitar, guitar–brain, and brain–brain), we calculated Pearson’s product correlation and multivariate Granger causality (GC) for amplitude and frequency modulations of guitar sounds and corresponding HB-HFN topology changes within the two performed music pieces: *Libertango* and *Comme un tango*. In addition, we investigated the behavior or robustness of the HB-HFN and the role of different types of network connections upon simulated gradual elimination of these connections in 15 5%-steps, both within the whole HB-HFN and within individual guitarists’ brains. We examined how this loss of connections or link removal changes network topology within the whole HB-HFN and the individual guitarists’ brains. Our expectation was that the behavior of the HB-HFN and the underlying NTD would remain relatively robust in response to the loss of connections, particularly if the loss only affected the brain of one guitarist.

## Methods

### Participants

A quartet of professional guitarists (Cuarteto Apasionado, Berlin) participated in the study (cf. Müller et al., 2018b). Participants’ mean age was 46.5 years (SD = 1.7). All participants (females) were right-handed and had been playing the guitar professionally for more than 35 years (mean = 37.8 years, SD = 1.3). The Ethics Committee of Max Planck Institute for Human Development approved the study, and it was performed in accordance with the ethical standards laid down in the 1964 Declaration of Helsinki. All participants volunteered for this experiment and gave their written informed consent prior to their inclusion in the study.

### EEG data acquisition and preprocessing

EEG measurement took place while the quartet played two music pieces: *Libertango* (Astor Piazolla) and *Comme un tango* (Patrick Roux). These musical pieces were chosen with regard to different aspects of interpersonal action coordination such as different phases of musical performance, consonant playing, changes of tempo, phases with different musical complexity, etc. The guitarists were positioned in an arc formation (refer to Supplementary Video S1 for visualization). EEG was simultaneously recorded using four electrode caps with 28 Ag/AgCl EEG active electrodes each, placed according to the international 10–10 system, with the reference electrode at the right mastoid and the ground electrode at the AFz position. Vertical and horizontal electrooculogram (EOG) was recorded to control for eye blinks and eye movements. The sampling rate was 5,000 Hz. Recorded frequency bands ranged from 0.01 to 1,000 Hz. All amplifiers (BrainAmps MR and BrainAmps ExG from Brain Products, Gilching, Germany) were connected to the same computer through PCI interfaces and synchronized by using BrainVision recorder software. Through one microphone each, the sounds of the guitars were recorded on four ExG channels, simultaneously with the EEG recordings. In addition, video and sound were recorded using a video camera connected to EEG computer through FireWire socket and Video Recorder as a component of BrainVision software (Brain Products, Gilching, Germany), synchronized in this way with EEG data acquisition. Data were re-referenced offline to an average of the left and right mastoid separately for each participant. Eye movement correction was accomplished by independent component analysis (Vigário, 1997). Thereafter, artifacts from head and body movements were rejected by visual inspection. Spontaneous EEG activity was resampled at 1000 Hz and divided into 5-s epochs. Event markers were set by a professional musician and correspond to different musical situations. The list of the events and their short description for both music pieces is presented in Supplementary Table S1. There were 10 and 14 segments in *Libertango* and *Comme un tango*, respectively, that were free of artifacts for all four guitarists. Further, to calculate the phase coupling, we used a moving time window approach with a window width of 500 ms and a time delay of 50 ms. A total of 91 time windows were captured using this moving time window approach.

### Phase synchronization (coupling) measures

Our analyses were conducted in a data-driven, directed, and frequency-resolved manner. To investigate phase synchronization within and between the frequencies, we applied an analytic complex-valued Morlet wavelet transform to compute the instantaneous phase in the frequency range from 2.5 to 60 Hz for nine different frequencies of interest (FOI): 2.5, 5, 10, 15, 20, 25, 30, 40, and 60 Hz. It is worth noting that both the choice of FOI and the parameter for the moving time window approach were selected to enable comparison with previous analyses. Furthermore, the FOI are in specific integer ratios to each other (e.g., 1:2, 1:3, 2:3, 1:4, 2:5, etc.) to ensure consistent analysis of CFC. The complex mother Morlet wavelet, also called Gabor wavelet, has a Gaussian shape around its central frequency *f*:


(1) $$w\left(t,f\right)={\left(\sigma^{2}\pi \right)}^{-1/4}e^{\left(\left(-t^{2}/2\sigma^{2}\right)+3/2\mathrm{\pi jft}\right)},j=\sqrt{-1}$$

in which σ is the standard deviation of the Gaussian envelope of the mother wavelet. The wavelet coefficients were calculated with a time step of 5 leading to a time resolution of 5 ms and frequency resolution of 0.5 Hz. To identify the phase relations between any two channels within and between the frequencies, a generalized phase difference (ΔΦ) was used to calculation of within- and cross-frequency coupling:


(2) $$\Delta \Phi \left(t\right)=n•\phi_{m}\left(t\right)-m•\phi_{n}\left(t\right)$$

where *m* and *n* are integers, and *ϕ_m,n_* are phases of two oscillators. In the case of WFC with *ϕ_m_* = *ϕ_n_*, the phase difference ΔΦ is calculated in the same way by setting *m* = *n* = 1. WFC and CFC within and between brains were determined using the *adaptive Integrative Coupling Index* (*aICI*) algorithm described in our previous study (Müller and Lindenberger, 2014), which allowed us to calculate this coupling index depending on the angle of phase differences determined in a given time window. In other words, *aICI* no longer reflect *in-phase* synchronization, where the angle of phase differences is close to zero, but is suitable for the determination of phase coupling at any chosen or previously determined phase angles. For these purposes, the Phase Synchronization Index (*PSI*) was determined first. It is defined by Müller et al. (2013):


(3) $$PSI_{\Delta \Phi }\left(f_{m,n}\right)=\left|e^{j\Delta \Phi^{k}\left(f_{m,n}\right)}\right|,j=\sqrt{-1}$$

where $\Delta \Phi^{k}$ is the phase difference between the instantaneous phases of the two signals at the frequencies *f_m_* and *f_n_* across *k* data points in the segment. During calculation of the *PSI*, we not only determined the mean direction or the length of the vector but also the angle of this vector ($\theta_{\Delta \Phi }$) in the complex space:


(4) $$\theta_{\Delta \Phi }\left(f_{m,n}\right)=\arctan\left(\frac{\langle j\sin\Delta \Phi^{k}\left(f_{m,n}\right)\rangle }{\langle \cos\Delta \Phi^{k}\left(f_{m,n}\right)\rangle }\right),\;j=\sqrt{-1}$$

Given the estimates of the phase difference between two signals, it is possible to ascertain how long the phase difference remains stable in defined phase angle boundaries by counting the number of points that are phase-locked in a defined time window. So, we divided the range between *θ* − π/4 and *θ* + π/4 into two ranges and distinguished between positive and negative deviations from phase angle $\theta_{\Delta \Phi }$. Within a time window of 500 ms, we separately counted the number of phase difference points in the range between *θ* − π/4 and *θ* (negative deviations) and in the range between *θ* and *θ* + π/4 (positive deviations). Phase difference values beyond these ranges were considered as non-synchronization. Before counting, successive points in the defined range (between *θ* − π/4 and *θ* + π/4) with a time interval shorter than a period of the corresponding oscillation at the given frequency (*T_i_* = 1/*f_i_*) were discarded from the analysis. This cleaning procedure effectively eliminated instances of accidental synchronization. On the basis of this counting, we obtained several synchronization indices: (1) the *Positive Coupling Index*, *PCI*, or the relative number of phase-locked points in the positive range (between *θ* and *θ* + π/4); (2) the *Negative Coupling Index*, *NCI*, or the relative number of phase-locked points in the negative range (between *θ* − π/4 and *θ*); (3) the *Absolute Coupling Index*, *ACI*, or the relative number of phase-locked points in the positive and negative ranges (i.e., between *θ* − π/4 and *θ* + π/4); (4) the *adaptive Integrative Coupling Index*, *aICI*, calculated by the formula (Müller and Lindenberger, 2014):


(5) $$\mathrm{aICI}=\frac{PCI+ACI}{2\cdot ACI}\cdot \sqrt{PCI}$$

The *aICI* is an *integrative* coupling index integrating the positive and negative shifts in phase difference of two signals and indicating the dominance of the positive shift in phase difference related to the common or absolute coupling. The *aICI* is equal to 1 when all points are phase-locked and positive; if all phase-locked points are negative or are out of range, the *aICI* will approach 0. Thus, the *aICI* measure ranges between 0 and 1 and is asymmetric (*aICI*_AB_ ≠ *aICI*_BA_), indicating the relative extent of positive phase synchronization. Moreover, by using the framework of “The Virtual Brain” (TVB, www.thevirtualbrain.org), simulation results in our previous study showed that all three measures (*PSI*, *ACI*, and *ICI*) capture the intended coupling properties (Müller et al., 2013). We restrict the description of our study results to the *aICI* measure, which is the most informative due to its directionality.

It should be noted that inter-brain synchronization measures (e.g., *aICI*) are robust to the detection of spurious phase synchronization between individuals, i.e., where no volume conduction is possible. Spurious phase synchrony at each individual level can occur because of the volume conduction problem (Tognoli and Kelso, 2009) but using only 28 electrodes per person with larger distances between the electrodes considerably reduces such influences, if any.

### Network construction and graph-theoretical approach (GTA)

**Figure 2:**
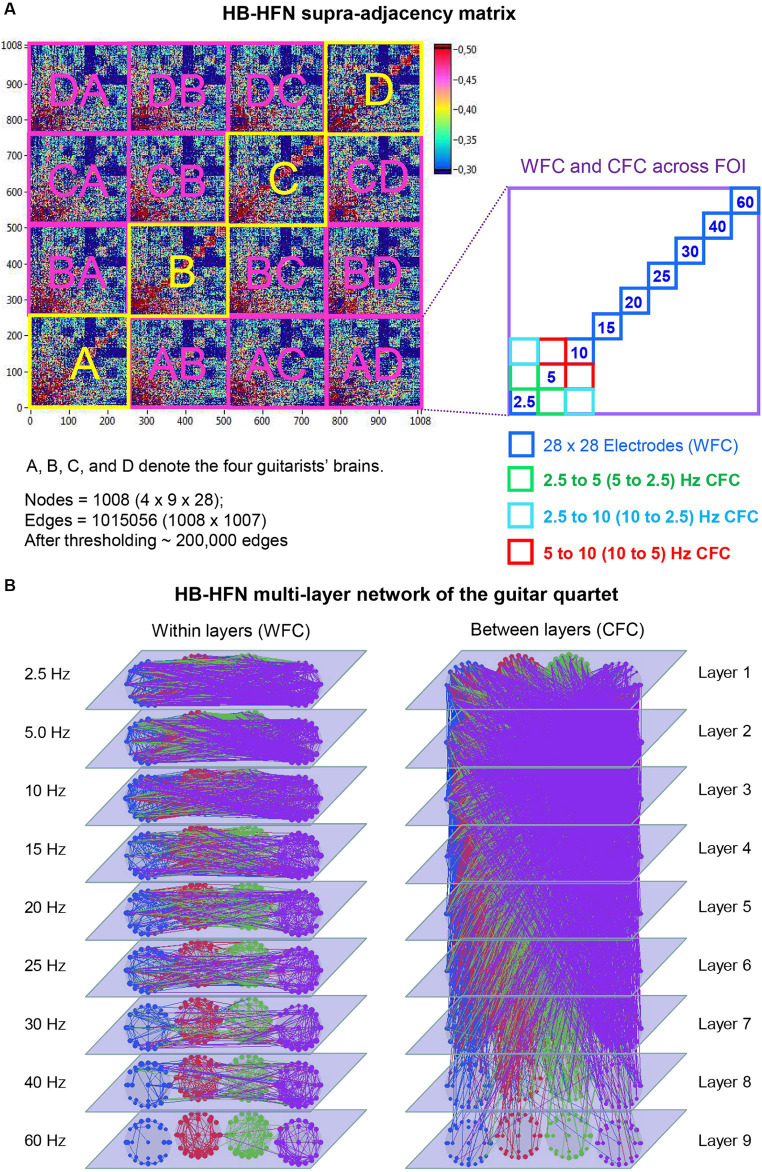
Representation of HB-HFN as a supra-adjacency matrix and a multilayer network. **(A)** HB-HFN supra-adjacency matrix. The supra-adjacency matrix (1,008 × 1,008) includes within-brain connectivity of the four guitarists (indicated in yellow) and between-brain connectivity (indicated in pink). The HB-HFN nodes comprise three components: guitarist’s brains (4), electrode sites (28), and oscillation frequency (9). As shown on the right, each guitarist’s brain (or a pair of brains for between-brain connectivity) consists of links between 28 electrodes within each of 9 frequencies (WFC) and between them (CFC). **(B)** HB-HFN multilayer network of the guitarist quartet. The within-layer communication (WFC on the left) and the between-layer communication (CFC on the right) are presented separately for visualization purposes. The 9 layers correspond to the 9 FOI. The predominance of low-frequency connections within and between the layers is evident here.

Using the aforementioned directed coupling index (*aICI*), we constructed a HB-HFN of the guitarist quartet during playing. This network comprises four brains with 28 electrodes each and 9 oscillation frequencies, and correspondingly includes coupling (WFC and CFC) within and between brains. Figure 2A represents this HB-HFN in form of a supra-adjacency matrix, where conventional GTA tools can be used to investigate the network properties. As already mentioned, this supra-adjacency matrix or the HB-HFN can be considered as a multilayer network, where WFC represents connections within each layer and CFC represents connections between the layers (see Figure 2B for details). This network comprises 1,008 nodes in total and more than 1 million edges if it is fully connected. For our analyses, we used thresholded networks with a connectivity threshold of 0.3, which was always higher than the significance level determined by the surrogate data procedure (*p* < 0.0001). At this threshold, the cost level of the networks (the ratio of the number of actual connections divided by the maximum possible number of connections in the network) was approximately 20%, corresponding to high sparsity of the resulting networks and allowing more accurate examination of the network topology. For the HB-HFN analyses, we used three well-known GTA measures capable to describe key network properties, including connectivity strength and the degree of the network segregation and integration.

#### Degrees and strengths

As *aICI* is a directed measure, we obtained the node in- and out-degrees, in which the in-degree is the sum of all incoming connections of node *i*, $k_{i}^{\mathrm{in}}=\underset{j\in N}{\sum }a_{ji}$, and the out-degree is the sum of all outgoing connections, $k_{i}^{\mathrm{out}}=\underset{j\in N}{\sum }a_{ij}$. To calculate strengths, we then replaced the sum of the links by the sum of the weights, $k_{i}^{w}=\underset{j\in N}{\sum }w_{ij}$, and calculated in- and out-strength, respectively. Overall strengths (*S*) are given by the sum of in- and out-strength. For statistical evaluation, we determined strengths for each node in the whole HB-HFN of the guitar quartet and then calculated them for WFC and CFC as well as for the within- and between-brain connections separately.

#### Clustering coefficient and characteristic path length

If the nearest neighbors of a node are also directly connected to each other, they form a cluster. For an individual node, the *CC_i_* is defined as the proportion of the number of pairs of *i*’s neighbors that are connected to the total number of pairs of *i*’s neighbors (see Figure 1B for details). In the case of a weighted directed graph the $CC_{i}^{wd}$ and the mean $CC^{wd}$are calculated as follows (Fagiolo, 2007):


(6) $$\begin{array}{l} CC^{wd}=\frac{1}{n}\underset{i\in N}{\sum }CC_{i}^{wd}=\\ \frac{1}{n}\underset{i\in N}{\sum }\frac{t_{i}^{wd}}{\left(k_{i}^{\mathrm{out}}+k_{i}^{\mathrm{in}}\right)\left(k_{i}^{\mathrm{out}}+k_{i}^{\mathrm{in}}-1\right)-2\sum_{j\in N}a_{ij}a_{ji}} \end{array}$$

with $t_{i}^{wd}=\frac{1}{2}\underset{j,h\in N}{\sum }{\left[\left({w_{ij}}^{1/3}{w_{ih}}^{1/3}{w_{jh}}^{1/3}\right)+\left({w_{ji}}^{1/3}{w_{hi}}^{1/3}{w_{hj}}^{1/3}\right)\right]}^{3}$ being the number of weighted directed triangles around a node *i*. The clustering coefficient is a measure of segregation.

Another important measure is the *CPL*. In the case of an unweighted graph, the shortest path length or distance *d_i,j_* between two nodes *i* and *j* is the minimal number of edges that have to be passed to go from *i* to *j* (see Figure 1B for details). This is also called the geodesic path between the nodes *i* and *j*. The *CLP* of a graph is the mean of the path lengths between all possible pairs of vertices (Watts and Strogatz, 1998):


(7) $$CPL^{wd}=\frac{1}{n}\underset{i\in N}{\sum }CPL_{i}^{wd}=\frac{1}{n}\underset{i\in N}{\sum }\frac{\sum_{j\in N,j\neq i}d_{ij}^{wd}}{n-1}$$

where $CPL_{i}^{wd}$ is the average shortest or characteristic path length from node *i* to all other nodes. In the case of a weighted and directed graph, the weight and direction of the links will be considered. *CPL* shows the degree of network integration, with a shorter *CPL* indicating higher network integration. Similar to strength, *CC* and *CPL* were calculated individually for each node, representing nodal measures (i.e., $CC_{i}^{wd}$ and $CPL_{i}^{wd}$).

### Relationships between the guitar sounds and the network topology measures

Further, we investigated the relationships between the guitar sounds and the HB-HFN topology indices. For these purposes, we first calculated amplitude and frequency modulations of the guitar sounds by Mean Power Frequency (*MPF*) and Envelope (*ENV*) for each of the guitar signals captured by microphones. The *MPF* was calculated by using the short-time Fourier transform (STFT) spectrogram from the biomedical LabVIEW tool and the MATLAB envelope function integrated LabVIEW was used for the *ENV* calculation. *MPF* and *ENV* underwent processing using the moving window approach (averaging within 500-ms time windows shifted by a 50-ms time delay), mirroring the methodology employed for the calculation of connectivity and topology indices in EEG signal analysis. To explore the associations between guitar sounds (i.e., *MPF* and *ENV*) and NTD indicated by temporal changes in different topology measures, we calculated (1) Pearson’s product correlation (*R*), reflecting linear relationships between the signals, and (2) multivariate Granger causality (*GC*), indicating causal or directional associations between the signals. For this calculation, the guitar sound and NTD data across the 91 time windows and 10 (Libertango) or 14 (Comme un tango) music sequences were collapsed together, thus providing a cascade-shaped time series of 910 (91 × 10) or 1,274 (91 × 14) data points for Libertango and Comme un Tango, respectively. For this analysis, the NTD indices were consistently averaged for each guitarist separately. In this way, we investigated the guitar–guitar, guitar–brain, and brain–brain relationships between guitarists (or even guitar-brain relationships within a guitarist).

### Robustness of HB-HFNs by stepwise elimination of different types of edges

To assess the robustness of HB-HFNs and elucidate the role or significance of network connections, we systematically manipulated the loss of various connection types within the entire HB-HFN and within individual guitarists’ brains. Our investigation focused on understanding how the removal of connections impacted the network topology both across the entire HB-HFN and within individual guitarists’ brains. This process involved a gradual elimination of connections in 15 5%-steps, utilizing three distinct types of removal: elimination of the weakest, strongest, and random connections. Subsequently, we computed the network topology at each manipulation step. For statistical evaluation, we determined the means and 95% confidence intervals (CIs) of the corresponding network topology indices at each step of the manipulation process. This comprehensive analysis sheds light on the robustness of HB-HFNs and provides insights into the dynamic role played by various types of connections in shaping the overall network topology.

### Statistical analysis

The three nodal measures (*S*, *CC*, and *CPL*) were initially determined for each time window and subsequently averaged across them within each music sequence. As mentioned above, the network nodes are a composition of three components: guitarist’s brain, electrode site, and oscillation frequency. Individual electrodes were grouped into three electrode sites: F (frontal – Fp1, Fpz, Fp2, AF7, AF8, F7, F3, Fz, F4, F8), C (central – T7, C5, C3, Cz, C4, C6, T8), and P (parietal – P7, P3, Pz, P4, P8, PO7, POz, PO8, O1, Oz, and O2). For the statistical evaluation of HB-HFN properties, nodes were regarded as cases that vary on three between-subject factors: Guitarist (A, B, C, and D), Site (F, C, and P), and Frequency (f1, f2, f3, f4, f5, f6, f7, f8, and f9). The music sequences of Libertango (10 sequences) and Comme un tango (14 sequences) were treated as within-subject factors in mixed ANOVAs. Further, to investigate the within- and between-brain (wB and bB, respectively) WFC and CFC, we summed up the couplings within these four groups of interest and collapsed the music sequences by averaging them for Libertango (MP1) and Comme un tango (MP2), respectively. We then conducted separate mixed ANOVAs for WFC and CFC, incorporating three between-subject factors as before and two within-subject factors: Coupling (wB and bB) and Music Piece (MP1 and MP2). When necessary, Greenhouse–Geisser epsilons were employed in all ANOVAs for nonsphericity correction. The Scheffé test was utilized for post-hoc testing of condition or network property differences. All statistical analyses were carried out using IBM SPSS Statistics 23.0 (SPSS Inc., Chicago, IL).

## Results

**Figure 3:**
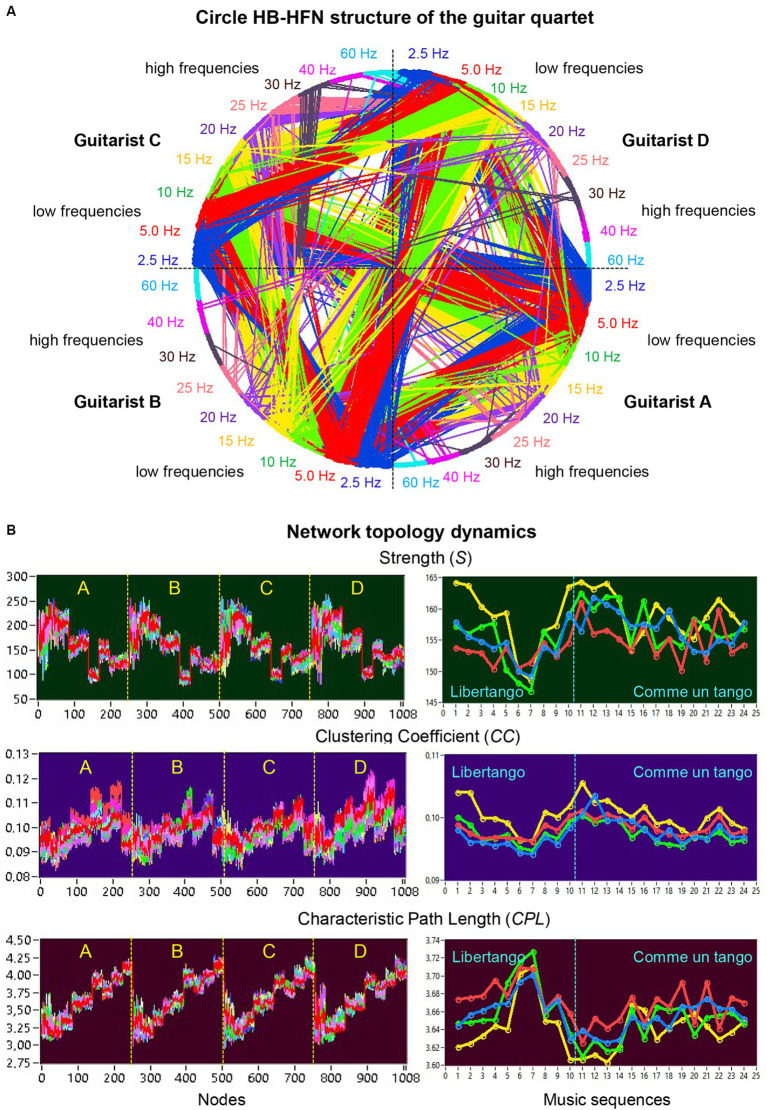
Circle HB-HFN structure of the guitar quartet and network topology dynamics. **(A)** Circle HB-HFN structure of the guitar quartet. The network nodes are arranged clockwise, starting from guitarist A (middle-right). Different frequencies (FOI) are represented by color. The predominance of low-frequency connections is also evident here (cf. Figure 2B). **(B)** Network topology dynamics. On the left, *S*, *CC*, and *CPL* are presented across the 1,008 nodes for 24 music sequences, indicated by color. On the right, the same GTA measures, averaged separately for the four guitarists’ brains (guitarist A in blue, guitarist B in red, guitarist C in green, and guitarist D in yellow), are depicted across the 24 music sequences: 1–10 for Libertango and 11–24 for Comme un tango.

Figure 3A shows an HB-HFN in the form of a circle, with the nodes arranged clockwise, and illustrates the relationships between different guitarists, electrode sites, and frequencies. It can be seen that the four guitarists mainly communicate with each other using the low frequencies. The network topology metrics determined in HB-HFNs at different time windows were first averaged over the different time windows within the music sequences. Figure 3B illustrates the network topology dynamics indicated by *S*, *CC*, and *CPL* across the 1,008 nodes over the 24 music sequences (left) and average values of the four guitarists across the music sequences of Libertango (10 sequences) and Comme un Tango (14 sequences). The Supplementary Video S2 features a 5-s music sequence from Libertango, providing a real-time display of connectivity changes in the HB-HFN throughout this duration.

### Network structure and topology dynamics across music sequences

**Figure 4:**
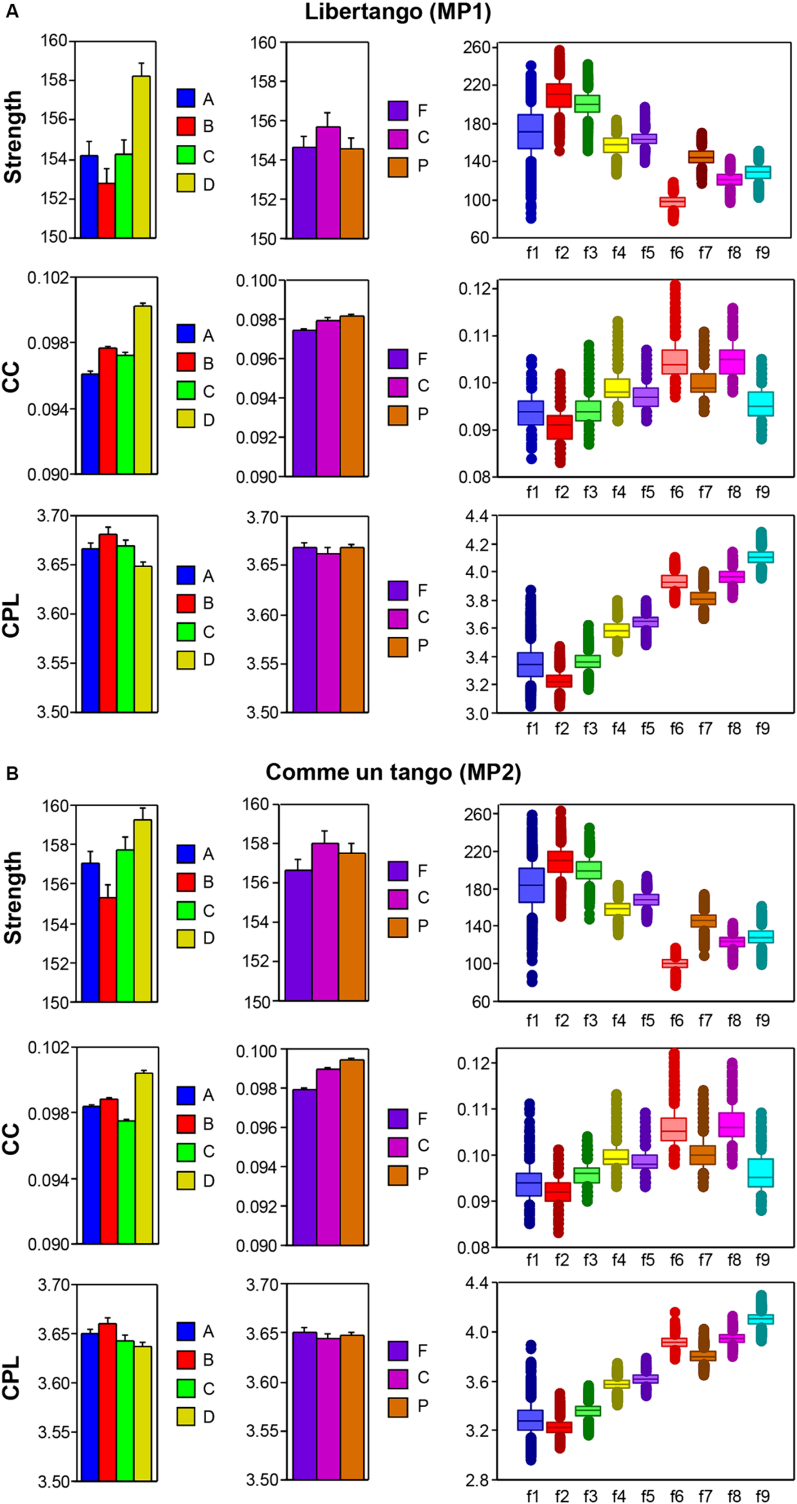
ANOVA results for mean values of the three GTA measure (S, CC, and CPL) for Libertango and Comme un tango, respectively. **(A)** ANONA results for Libertango. Main effects of the factors Guitarist (A, B, C, and D), Site (F, C, and P), and Frequency (f1-f9) are presented here. **(B)** ANONA results for Comme un tango. The same main effects as in **(A)** are presented here. The main effects of the factor Sequence for both music pieces can be obtained in Figure 3 presented for the four guitarists.

The nodal network topology indices (*S*, *CC*, and *CPL*) determined in the HB-HFN and averaged over the different time windows were analyzed using mixed ANOVAs with the three between-subject factors Guitarist (A, B, C, and D), Site (F, C, and P) and Frequency (9 frequencies), which capture the HB-HFN structure, and one within-subject factor Sequence (10, respectively 14). To assess the dynamics within the music sequences, we determined not only the mean values over the time windows, but also the standard deviations (SDs) and subjected them to the same mixed ANOVAs. All ANOVAs for both mean values and SDs revealed significant main effects and significant interactions for all network metrics (all *Ps* < 0.001; see Supplementary material for details). The main effects for the factors Guitarist, Site, and Frequency are presented in Figures 4A,B for Libertango and Comme un Tango, respectively. Significant differences in the topology indices between the four guitarists apparently indicate different roles of the guitarists in the common network. Interestingly, guitarist D showed higher strength and *CC* as well as the shortest *CPL* in both music pieces, indicating her high segregation and integration in the common HB-HFN. Significant differences in the topology indices between the electrode sites mostly indicate the predominance of centro-parietal sites in the HB-HFN. It can also be seen that the strengths of nodes in the common HB-HFN decrease with high frequency, while *CC* and especially *CPL* increase (*CPL* becomes longer). All the changes across the frequencies are highly significant and indicate different contributions of these frequencies to network topology and functioning. As shown in Figure 3, the network topology also changes across sequences, indicating that the network topology is nonstationary and contingent on musical situation. These changes also vary across the four guitarists, indicating that the guitarists significantly change their impact on the quartet play. Moreover, significant interactions among all the factors indicate that the observed changes in the network topology are not absolute but are influenced by each other and are in permanent interplay.

**Figure 5:**
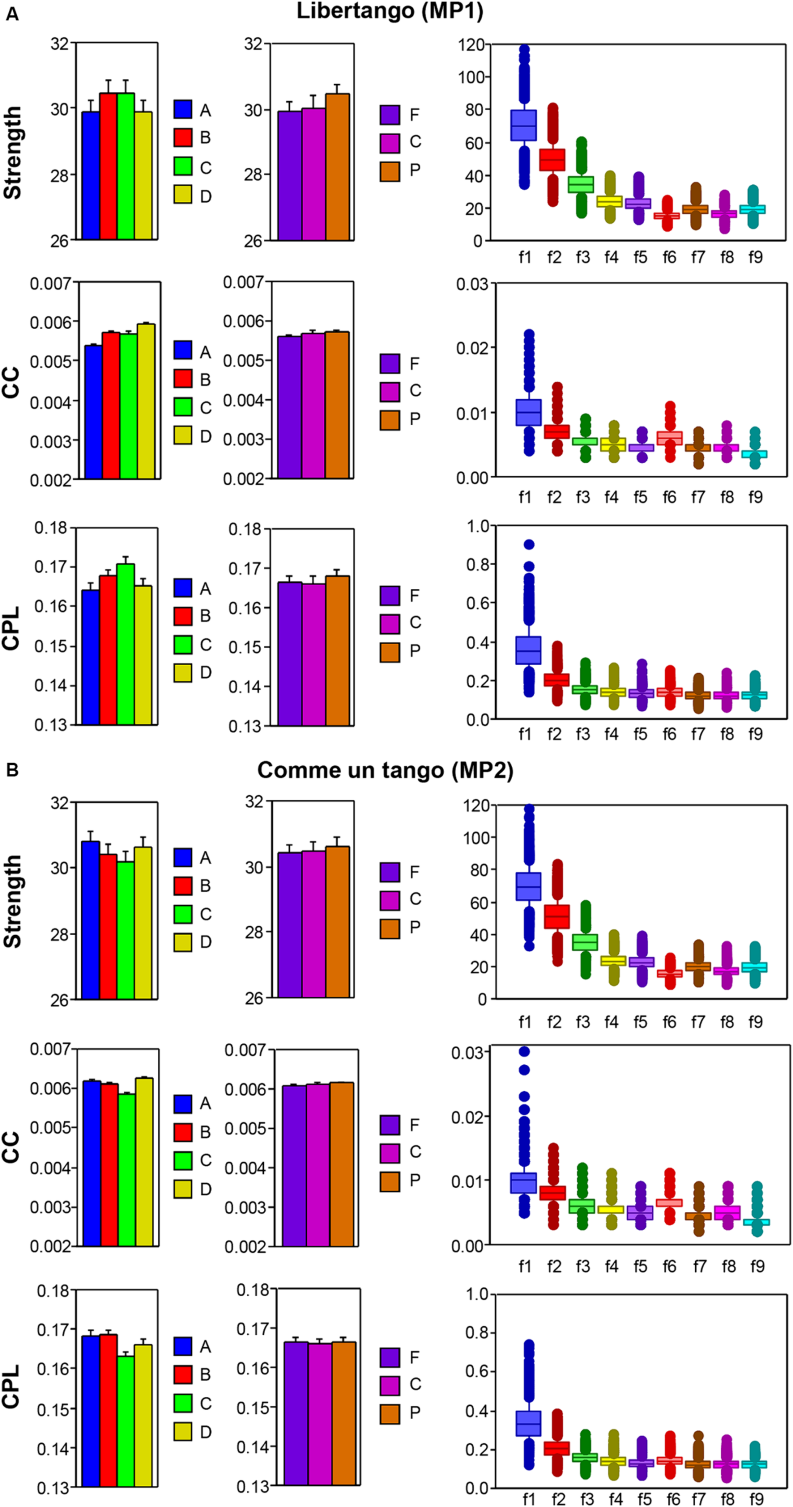
ANOVA results for SD values of the three GTA measure (S, CC, and CPL) for Libertango and Comme un Tango, respectively. **(A)** ANOVA results for Libertango. Main effects of the factors Guitarist (A, B, C, and D), Site (F, C, and P), and Frequency (f1–f9) are presented here. **(B)** ANOVA results for Comme un tango. The same main effects as in **(A)** are presented here. The main effects of the factor Sequence for both music pieces can be found in the Supplementary materials.

The main effect of the SD differences for the factors Guitarist, Site, and Frequency are presented in Figures 5A,B for Libertango and Comme un tango, respectively. It can be seen that the variability in the network topology determined by the SD differs among the four guitarists and also varies with the electrode sites, oscillation frequency, and music sequences in the two pieces of music (see Supplementary material for further details). Interestingly, despite the different changes in the network topology shown in Figure 4, the SD decreases with higher frequency for all topology metrics (see Figure 5).

#### WFC and CFC as well as intra- and inter-brain strengths

**Figure 6:**
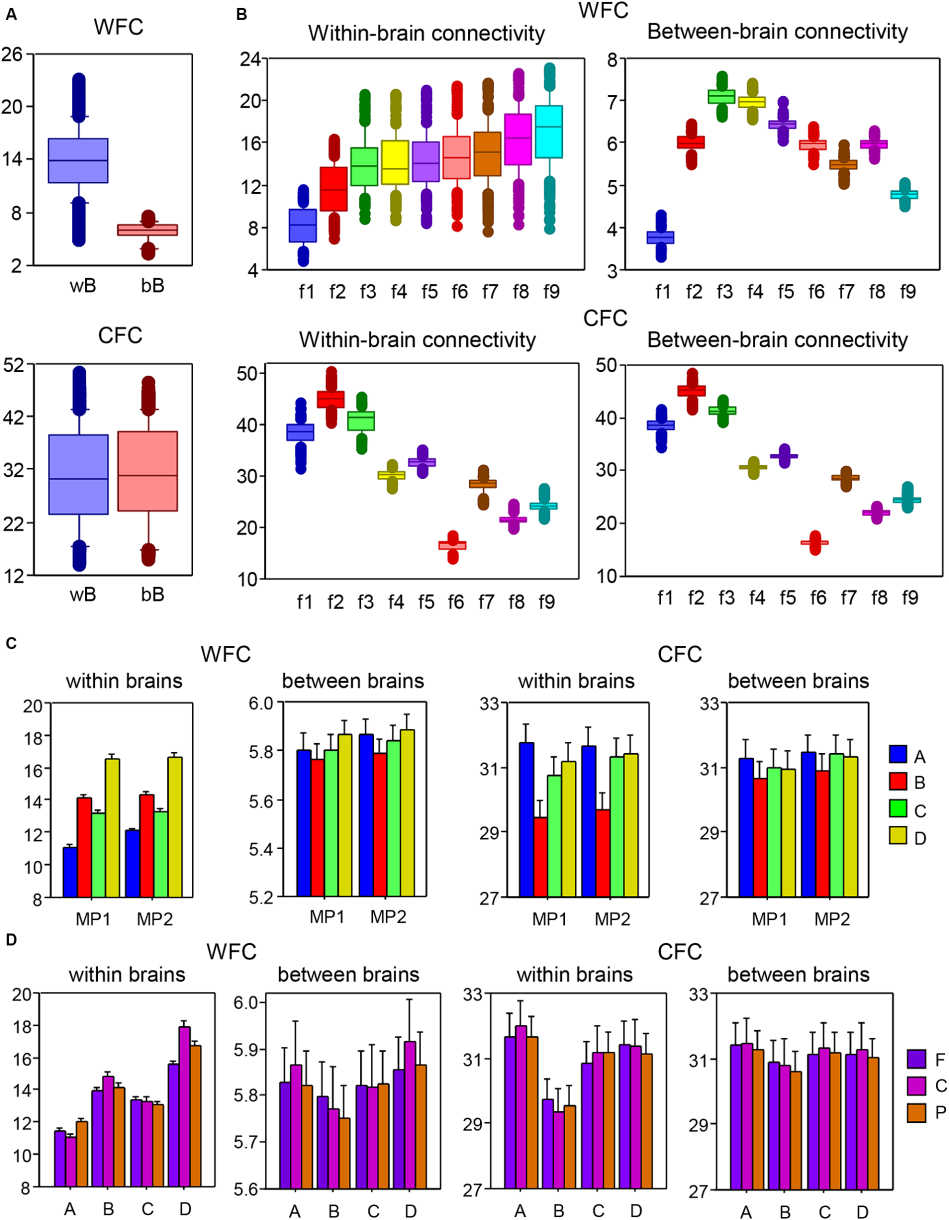
ANOVA results for mean values of within- and between-brain connectivity measured by WFC and CFC, respectively. **(A)** ANOVA results for the main effect of Coupling. The main effect of the factor Coupling (within vs. between brains) for WFC (top) and CFC (bottom) is presented in box plots. **(B)** ANOVA results for the main effect of Frequency. The main effect of the factor Frequency for WFC (top) and CFC (bottom) is presented in box plots. Note that within-brain (left) and between-brain (right) couplings are shown in two separate box plots. **(C)** ANOVA results for the interaction Guitarist by Music Piece (MP1: Libertango; MP2: Comme un tango). The interaction of Guitarist by Music Piece for WFC (left) and CFC (right) is presented for within-brain and between-brain couplings in two separate diagrams. **(D)** ANOVA results for the interaction of Guitarist by Site. The interaction of Guitarist by Site for WFC (left) and CFC (right) is presented for within-brain and between-brain couplings in two separate diagrams.

To investigate the coupling within and between layers in the multilayer HB-HFN, we determined WFC and CFC within and between brains separately for each node and averaged these across time windows and music sequences for Libertango and Comme un tango, respectively. WFC and CFC strengths were subjected to two separate mixed ANOVAs with three between-subject factors Guitarist, Site, and Frequency and two within-subject factors Music Piece (MP1 vs. MP2) and Coupling (within-brain vs. between-brain coupling). All main effects and most interactions were highly significant (all *P*s < 0.001, with some exceptions). Results of these ANOVAs are presented in the Supplementary materials. As shown in Figure 6A, the WFC or coupling within the layers was much stronger within brains than between brains, while CFC (or coupling between the layers) was significantly higher between brains than within them. Figure 6B shows that the coupling within the layers (WFC) increases with higher frequency and the coupling between the layers (CFC) decreases. The increase in WFC is primarily due to the within-brain coupling, WFC between the brains increases only up to 10 Hz and then gradually decreases. As shown in Figure 6C, the four guitarists showed different coupling patterns with high WFC in guitarist D and high CFC in guitarist A for both MP1 and MP2, respectively. This indicates that the coupling within and between the layers differs among these guitarists, especially within their brains. The coupling is also different in the two music pieces, with overall higher WFC and also CFC in MP2 than in MP1. Moreover, as shown in Figure 6D, the guitarists differ also in WFC and CFC as well as in their within- and between-brain coupling with respect to the topological distribution or brain sites (see Supplementary materials for more details).

### Network topology dynamics and its relationship to the guitar sounds

**Figure 7:**
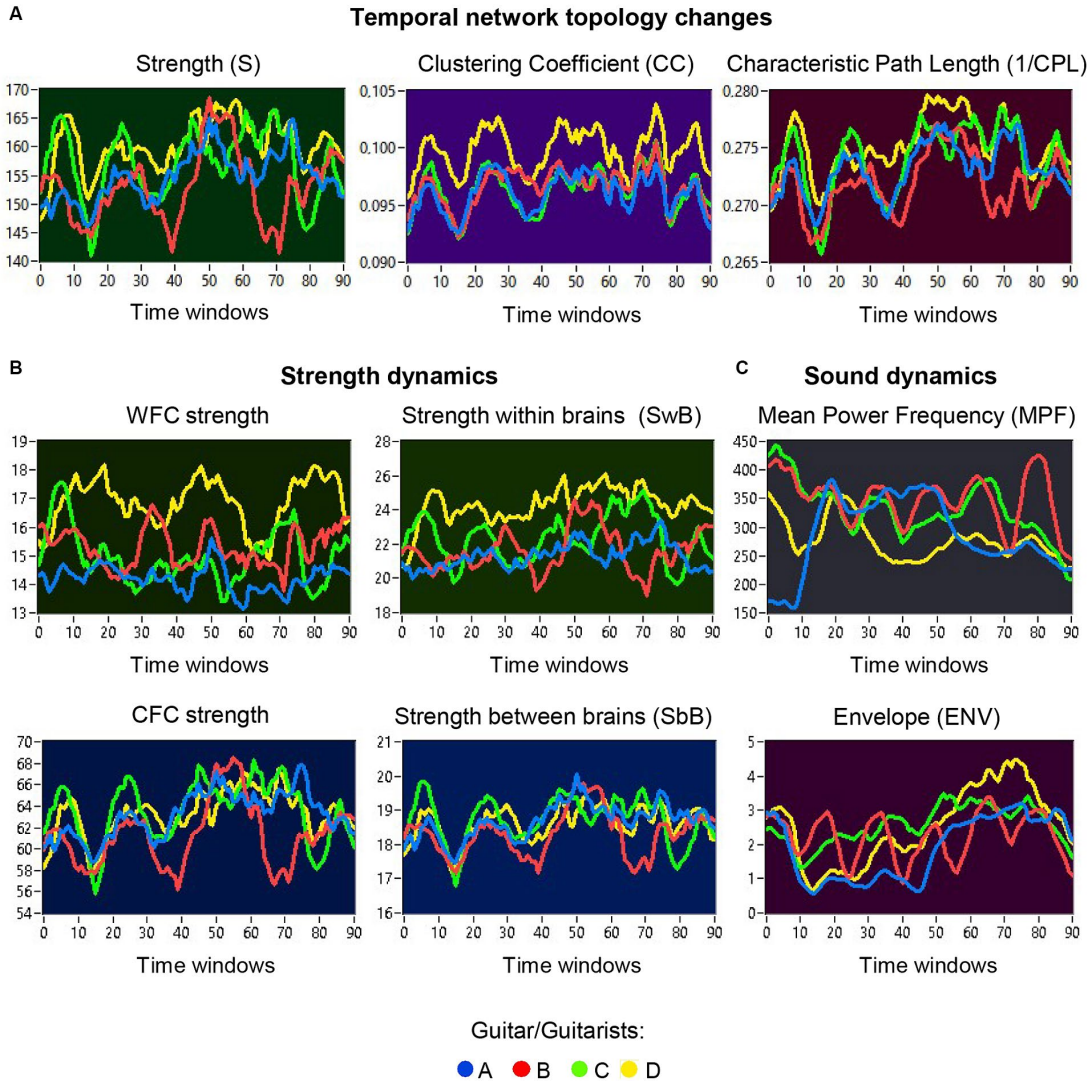
Temporal network topology changes and sound dynamics. **(A)** Examples of temporal changes in network topology within a music sequence, indicated by *S*, *CC*, and *CPL*. The temporal changes in the network topology are depicted separately for the four guitarists (guitarist A in blue, guitarist B in red, guitarist C in green, and guitarist D in yellow) across different time windows. **(B)** Examples of temporal changes in strengths within a music sequence, calculated separately for WFC and CFC, as well as for within- and between-brain coupling. The temporal changes in strengths are also depicted separately for the four guitarists across different time windows. **(C)** Examples of sound dynamics within a music sequence, indicated by *MPF* and *ENV*. Sound dynamics, as indicated by *MPF* (top) and *ENV* (bottom), are presented separately for the four guitar sounds (guitar A in blue, guitar B in red, guitar C in green, and guitar D in yellow) across different time windows.

**Figure 8:**
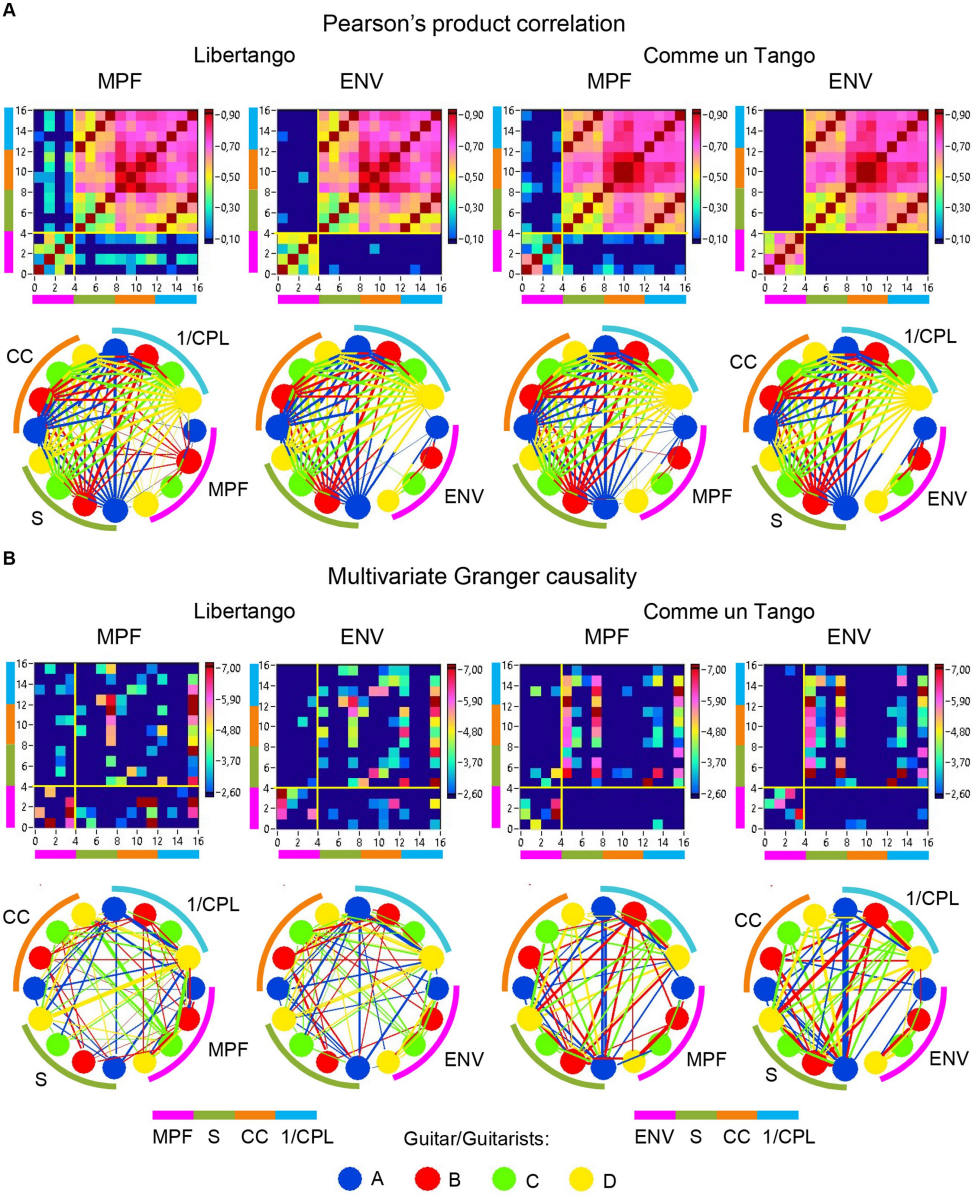
Linear and directional relationships between guitar sounds (*MPF* and *ENV*) and NTD indices (*S*, *CC*, and *1/CPL*) for Libertango and Comme un Tango, respectively. **(A)** Linear relationships indicated by Pearson’s product correlation. **(B)** Directional relationships indicated by multivariate Granger causality. The relationships are presented as matrices or heatmaps and circular connectivity maps. The different measures in the heatmaps and circular connectivity maps are highlighted by stripes or arcs of different colors: the pink stripe or arc indicates the four guitar sounds, determined by *MPF* or *ENV* (nodes 1–4), the green stripe or arc indicates the *S* in the four guitarists’ brains (nodes 5–8), the light brown stripe or arc indicates the *CC* in the four guitarists’ brains (nodes 9–12), and the light blue stripe or arc indicates the inverse *CPL* or *1/CPL* in the four guitarists’ brains (nodes 13–16). The four guitars or guitarists in the connectivity maps are indicated by color. The linear relationships are symmetric and the directional relationships are asymmetric. The direction of the links is coded by color. Note that the links in the directional connectivity maps are either unidirectional or bidirectional.

**Figure 9:**
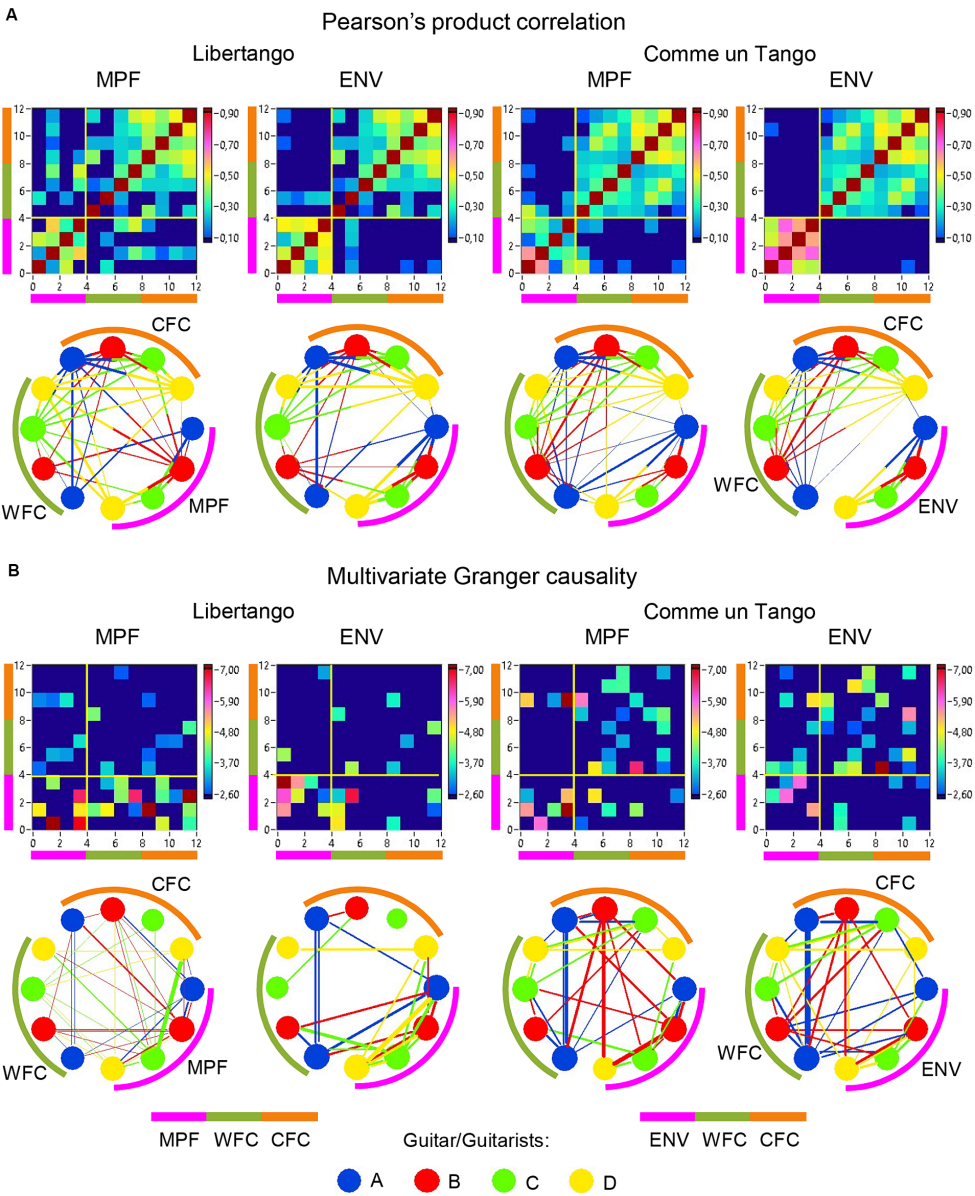
Linear and directional relationships between guitar sounds (*MPF* and *ENV*) and WFC and CFC strengths for Libertango and Comme un tango, respectively. **(A)** Linear relationships indicated by Pearson’s product correlation. **(B)** Directional relationships indicated by multivariate Granger causality. The relationships are presented as matrices or heatmaps and circular connectivity maps. The different measures in the heatmaps and circular connectivity maps are highlighted by stripes or arcs of different colors: the pink stripe or arc indicates the four guitar sounds, determined by *MPF* or *ENV* (nodes 1–4), the green stripe or arc indicates the WFC strengths in the four guitarists’ brains (nodes 5–8), and the light brown stripe or arc indicates the CFC strengths in the four guitarists’ brains (nodes 9–12). The four guitars or guitarists in the connectivity maps are indicated by color. The linear relationships are symmetric and the directional relationships are asymmetric. The direction of the links is coded by color. Note that the links in the directional connectivity maps are either unidirectional or bidirectional.

**Figure 10:**
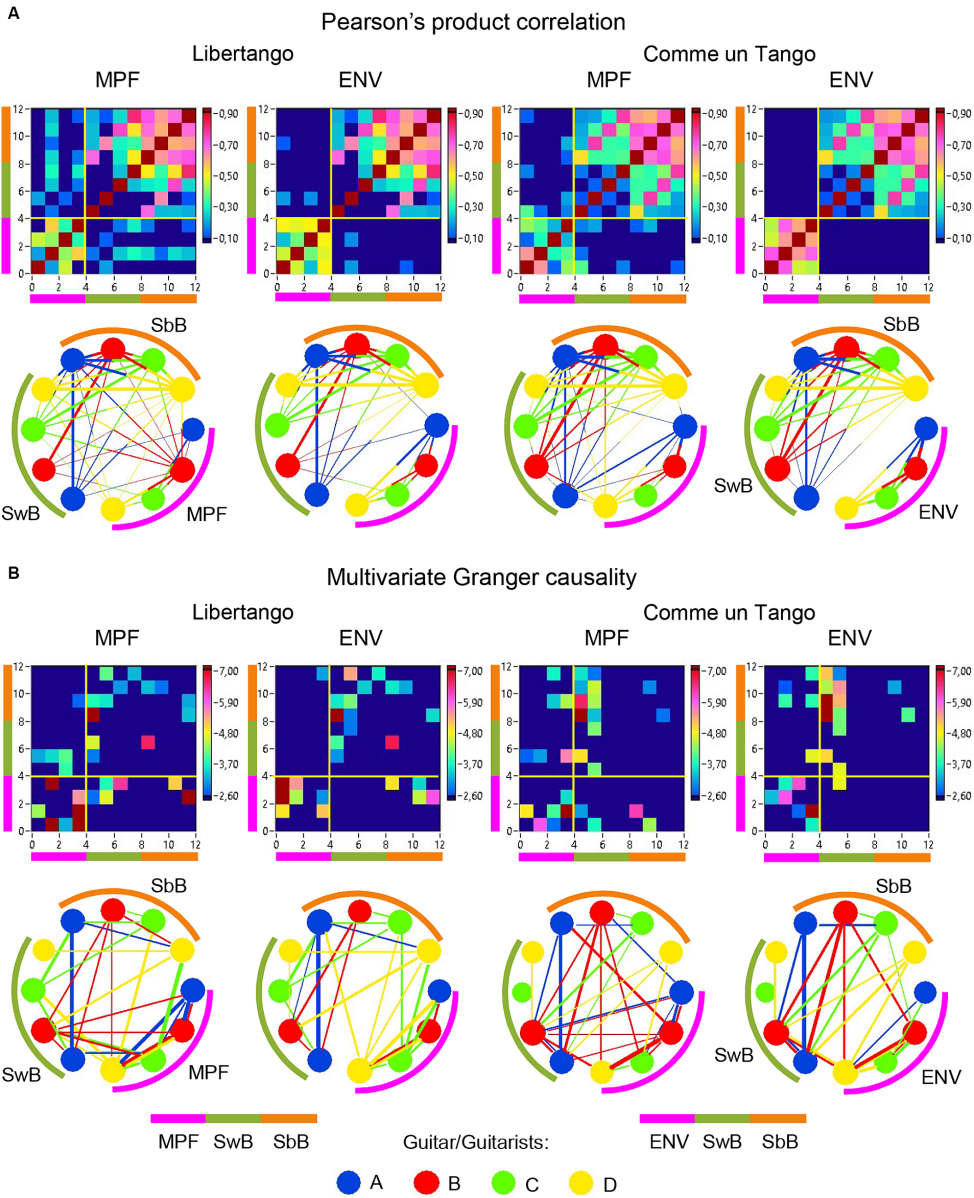
Linear and directional relationships between guitar sounds (*MPF* and *ENV*) and within- and between-brain strengths for Libertango and Comme un Tango, respectively. **(A)** Linear relationships indicated by Pearson’s product correlation. **(B)** Directional relationships indicated by multivariate Granger causality. The relationships are presented as matrices or heatmaps and circular connectivity maps. The different measures in the heatmaps and circular connectivity maps are highlighted by stripes or arcs of different colors: the purple stripe or arc indicates the four guitar sounds, determined by *MPF* or *ENV* (nodes 1–4), the green stripe or arc indicates the within-brain strengths (wB) in the four guitarists’ brains (nodes 5–8), and the light brown stripe or arc indicates the between-brain strengths (bB) in the four guitarists’ brains (nodes 9–12). The four guitars or guitarists in the connectivity maps are indicated by color: guitar/guitarist A in blue, guitar/guitarist B in red, guitar/guitarist C in green, and guitar/guitarist D in yellow. The linear relationships are symmetric and the directional relationships are asymmetric. The direction of the links is coded by color. Note that the links in the directional connectivity maps are either unidirectional or bidirectional.

The previous analyses have shown that the network topology metrics exhibit a certain variability. Here we aim to examine whether this variability or underlying dynamics in network topology is related to the amplitude and frequency modulations of guitar sounds. For these purposes, we calculated two different characteristics of guitar sounds of the four guitarists (*MPF* and *ENV*) and related them to the HB-HFN topology metrics averaged for each guitarist’s brain. In these analyses, instead of the *CPL*, we used its inverse (1/*CPL*), to obtain the same direction of changes as other topology measures. These dynamics are exemplarily presented in Figure 7. To investigate the relationships between all these signals, we calculated for each of the pieces of music: (1) Pearson’s product correlation (*R*), reflecting linear relationships between the signals, and (2) multivariate Granger causality (*GC*), indicating causal or directional associations between the signals. Figure 8 shows the relationships between the guitar sound characteristics (*MPF* and *ENV*) and the three HB-HFN measures (*S*, *CC*, and inverse *CPL*). It can be seen that the linear relationship determined by the Pearson’s product correlation is relatively strong between the four musician’s guitar sounds and especially between NTD indices. The correlation between the guitar sounds and the NTD is moderate for MPF signals, especially during Libertango. The multivariate Granger causality also shows specific relationships between the guitar sounds and between the NTD indices but also between the sounds and NTD, particularly during Libertango. Most interestingly, the last relationships are mostly unidirectional and mostly go from guitar sounds to NTD indices. In other words, guitar sounds affect or influence the hyper-brain communication more strongly than vice versa. Figure 9 displays the relationships between the guitar sounds and the coupling within (WFC) and between (CFC) the HB-HFN layers. As to be seen, the four guitarists are more similar in terms of inter-layer or CFC communication as compared to the intra-layer or WFC communication. *MPF* shows stronger relationships with NTD, especially with respect to within-layer coupling or WFC and especially during Libertango, also mostly directed from sounds to the HB-HFN coupling. Figure 10 illustrates the relationships between guitar sounds and the intra- and inter-brain couplings. As expected, the inter-brain couplings of the four guitarists are strongly related to each other in the case of a linear relationship (correlation) but practically disappear (with some exceptions) in the case of multivariate GC because of the absence of clear directional connections. Interestingly, there are relatively strong correlations between intra- and inter-brain coupling strength in each of the guitarists. In other words, high intra-brain strengths in a guitarist are strongly related to the connection strengths from this guitarist to all others. In the case of multivariate GC, this relationship, if present, is mostly unidirectional and goes from inter-brain to intra-brain strength, and may involve not only the same, but also the other guitarists (see Figure 10 for details). There are several connections between guitar sounds and strengths (both intra- and inter-brain) mostly going from guitar to brain, especially during Libertango, but also from brain to guitar, especially during Comme un tango.

### Robustness of HB-HFNs by stepwise elimination of different types of edges

**Figure 11:**
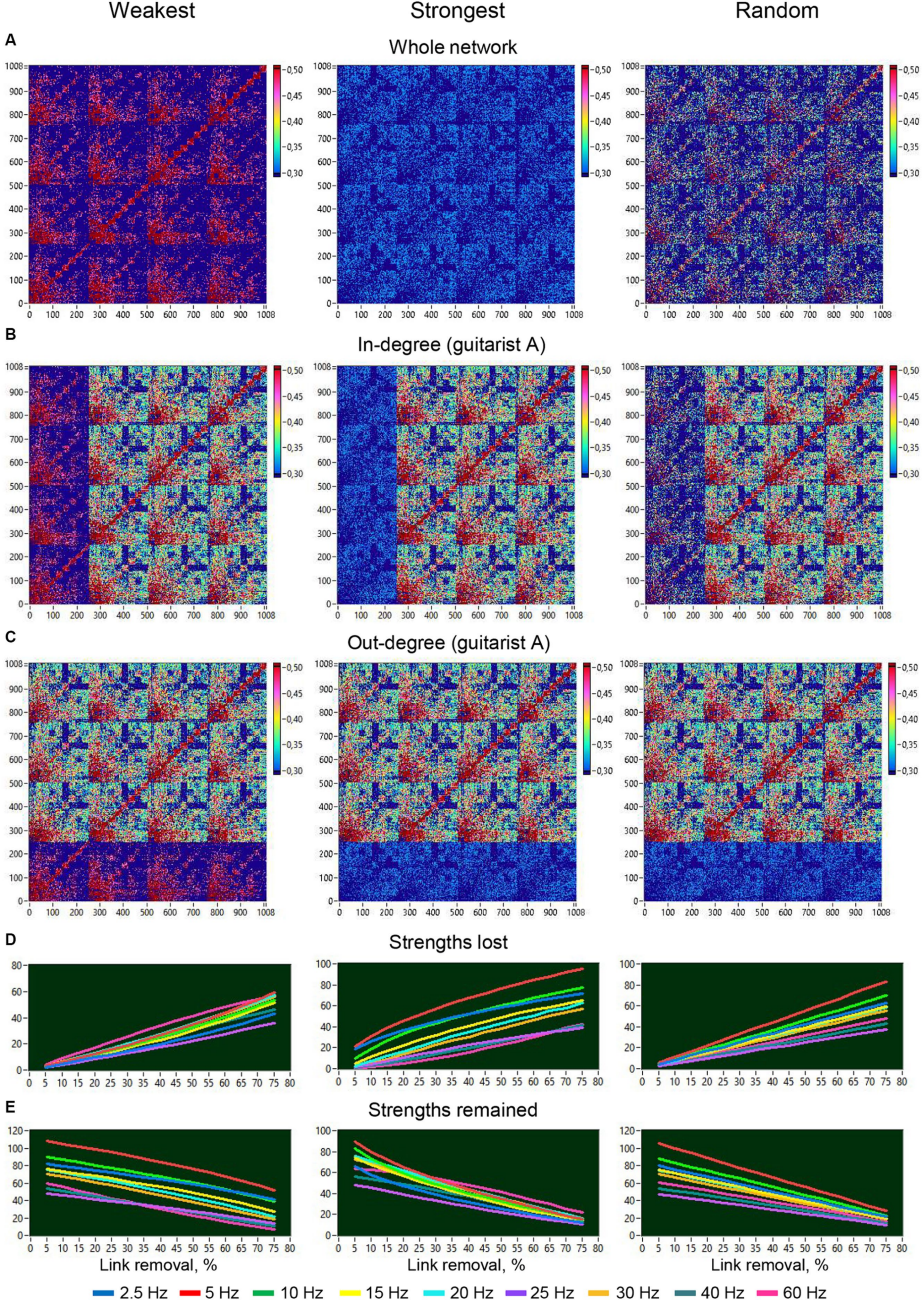
Removal of different types of links from the entire network or from one guitarist’s brain and changes in strengths of different frequencies as a function of this removal. **(A)** Removal of different types of links from the entire network. **(B)** Removal of different types of links or in-degree manipulation in guitarist A. **(C)** Removal of different types of links or out-degree manipulation in guitarist A. **(D)** Changes in loss of strengths at different frequency nodes as a function of link removal from the entire network. **(E)** Changes in retaining strengths at different frequency nodes as a function of link removal from the entire network.

To investigate the robustness of the HB-HFN and the role of network connections, we manipulated the loss of the different types of connections within the whole HB-HFN and within individual guitarists’ brains and examined how this loss of connections changes the network topology both within the whole HB-HFN and in individual guitarists’ brains. To do so, we gradually eliminated connections in 15 5%-steps with three different types of loss, of the weakest, the strongest, and of random connections, and calculated the network topology each time. We compared these changes with the network topology without loss of connections. Figures 11A–C depict the respective manipulations for a 75% loss across the entire network and specifically for the in- and out-degree of guitarist A. Figures 11D,E illustrate the dynamics of both lost and retained strengths throughout the 15 elimination steps for the 9 FOI. The removal of the weakest connections involves both high and low-frequency connections or nodes, but the low-frequency connections with high strengths are consistently preserved throughout all 15 elimination steps. The removal of the strongest connections mainly impacts the low-frequency connections, and the preservation of these connections and their strength rapidly decreases across the 15 elimination steps. Interestingly, when the connections are removed randomly, the low-frequency connections are increasingly affected, and the preservation of these connections also decreases at a high rate, but they persist throughout all elimination steps.

**Figure 12:**
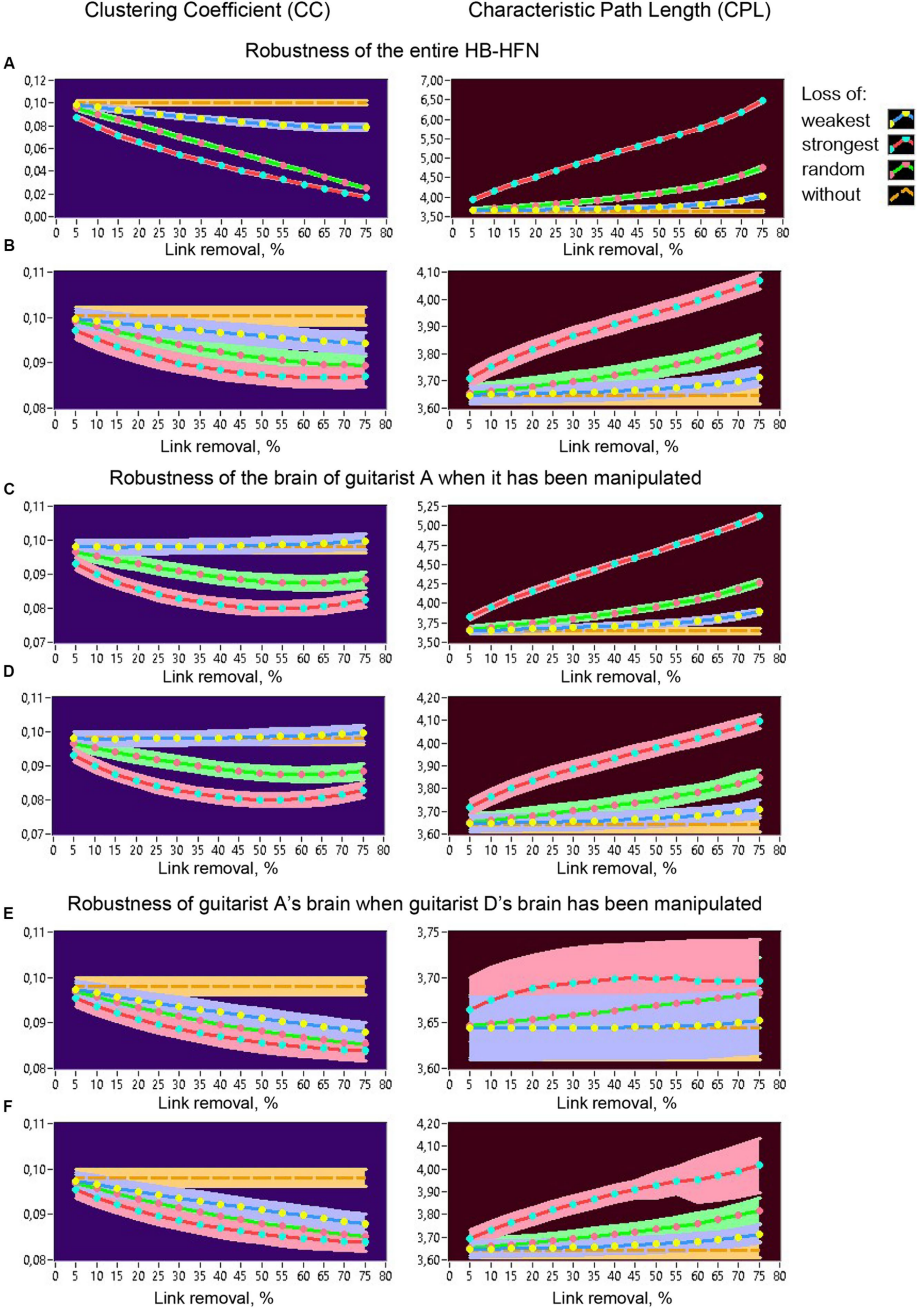
Robustness of the entire HB-HFN and of an individual guitarist’s brain indicated by changes in *CC* and *CPL* as a function of link removal of different types. **(A)** Robustness of the entire HB-HFN when the entire HB-HFN has been manipulated. **(B)** Robustness of the entire HB-HFN when links have been removed only in one guitarist’s brain (here guitarist A). **(C)** Robustness of one guitarist’s brain (here guitarist A) as a part of the HB-HFN when in-degree in the same guitarist has been manipulated. **(D)** Robustness of the one guitarist’s brain (here guitarist A) as a part of the HB-HFN when out-degree in the same guitarist has been manipulated. **(E)** Robustness of one guitarist’s brain (here guitarist A) as a part of the HB-HFN when in-degree in another guitarist (here guitarist D) has been manipulated. **(F)** Robustness of the one guitarist’s brain (here guitarist A) as a part of the HB-HFN when out-degree in another guitarist (here guitarist D) has been manipulated. Changes in *CC* (left) and *CPL* (right) as a function of link removal across the 15 5%-steps are presented in all diagrams for different types of link removal: loss of weakest, strongest, and random connections, in comparison to without removal.

All topology measures were averaged across the time windows within a music sequence and mean values (+/-CI) are presented in the diagrams for different types of connection loss compared to no loss. Figure 12A shows changes of *CC* (left) and *CPL* (right) in the whole HB-HFN when the loss of connections was also manipulated in the entire network. As expected, *CC* decreases and *CPL* increases or becomes longer as connection loss increases, especially when the strongest or random connections are lost. Importantly, *CPL* remains relatively robust when the weakest (and partly also random) connections are lost, while *CC* decreases significantly even when the weakest connections are lost. Figure 12B shows the changes in network topology in the entire HB-HFN when the loss of connections was simulated only in the brain of one guitarist (here guitarist A) with regard to the in-degree. Since the out-degree manipulation showed similar results in terms of network topology changes across the entire HB-HFN, it is not shown here. It can be seen that NTDs undergo similar changes as before, but the extent of these changes is much smaller. Most importantly, the topology mostly does not change significantly when the strongest connections of a guitarist are retained. Only when the strongest (or even random) connections are lost in one of the guitarists do the changes in the NTD become significant, especially in the *CPL*, which is apparently less robust than the *CC*, although the changes in the *CC* are also significant. In Figures 12C–F, the robustness within an individual brain (here guitarist A) is displayed when the loss of connections is manipulated in the same guitarist’s brain (Figures 12C,D) or in the other guitarist’s brain (here guitarist D; Figures 12E,F). When the loss of connections is manipulated in the same guitarist’s brain, *CC* does not change at all when the strongest connections remain, and it decreases non-linearly when the strongest or random connections are lost, regardless of whether the in-degree or the out-degree has been manipulated (Figures 12C,D). The *CPL* in this case becomes longer, especially when the in-degree is manipulated, and especially when the strongest connections are lost. If the strongest connections are retained, the functionality of the individual subnetwork is largely preserved. When the loss of connections in the brain of the other guitarist (here guitarist D) is manipulated, *CC* in the brain of guitarist A decreases for all types of manipulations (especially when the strongest connections are lost), regardless of whether the in-degree or the out-degree was manipulated (Figures 12E,F). The *CPL* in this case does not change significantly when the in-degree of guitarist D is manipulated, while it increases or becomes longer when the out-degree of guitarist D is manipulated or decreases, especially when the strongest connections are lost. Similar results of network topology changes in relation to guitarist D can be found in the Supplementary materials, which indicate invariance of topology changes with respect to different guitarists.

## Discussion

The primary objective of this study was to investigate the multilayer hyper-brain network dynamics and architecture in a quartet of guitarists playing together, where the WFC indicates coupling within the layers and the CFC indicates coupling between them. The main findings are that: (a) the four guitarists significantly differ in their network topology dynamics, apparently indicating their different roles in the common hyper-brain network during play; (b) hyper-brain coupling strengths involving the four guitarist brains decrease with higher oscillation frequency, while *CC* and especially *CPL* increase with ascending frequency (*CPL* becomes longer); (c) the couplings within (WFC) and between (CFC) the layers as well as within and between brains differ with respect to the guitarists, oscillation frequency, brain sites, and the two music pieces, with generally higher WFC within the brains and higher CFC between the brains; (d) the variability of all NTD measures exponentially decreases with higher oscillation frequency, indicating high variability of low-frequency nodes in HB-HFN; (e) different NTD measures show linear and causal relationships with guitar sounds, varying in amplitude (*ENV*) and frequency (*MPF*) characteristics, with the guitar sounds having a stronger influence on the brain’s NTD than vice versa; (f) the HB-HFN behavior and underlying NTD are relatively robust against the loss of connections, especially when the strongest connections are preserved and especially when connection loss only affects the brain of one guitarist.

As suggested, significant differences in the topology indices among the four guitarists apparently indicates that the guitarists have different roles in the common HB-HFN, with guitarist D characterized by high segregation and high integration of coupling in the common HB-HFN. Furthermore, the differences between guitarists vary depending on the oscillation frequency and brain regions, and most importantly, the network topology of guitarists differs in different music sequences and pieces of music, indicating that the network topology is nonstationary and contingent on musical situation. Moreover, these differences between guitarists also depend on the couplings type (WFC or CFC) and their properties (within or between the brains). The dependence of the guitarists’ network topology on the musical situation as well as on coupling properties has also been demonstrated in our previous work (Sänger et al., 2012, 2013; Müller et al., 2013, 2018b; Müller and Lindenberger, 2019).

Ensemble performance has been conceived as a microcosm of social interaction in which the ensemble functions as a dynamic system and the individual musicians as processing units (D’Ausilio et al., 2015). We show here that this microcosm has a multi-layered structure and the musicians act differently at different organizational levels in terms of their neural connections, which collectively form the entire network and its constituent parts. It can also be seen that the strengths of nodes in the common HB-HFN decrease with high frequency, while *CC* and especially *CPL* increase. All the changes across the frequencies forming different layers in multilayer networks are highly significant and indicate different contributions of these frequencies to the network topology and functioning, with lower frequencies contributing to network integration (indicated by shorter *CPL*) and higher frequencies providing or causing network segregation (indicated by higher *CC*) (Müller et al., 2018b). Interestingly, there is a discrepancy regarding WFC with respect to changes of within- and between-brain coupling with advancing frequency: while the WFC within brains increases with higher frequency, WFC between the brains increases only up to 10 Hz and then gradually decreases. Similar coupling patterns were also found previously (Müller et al., 2013, 2018b). However, we show here that these patterns are characteristic only for the WFC, while CFC (both within and between brains) practically showed strong decrease with growing frequency. This indicates that CFC at low frequencies is of paramount importance with respect to neuronal integration between different network structures and network layers. Similar results were found in a kissing study, where WFC and CFC were used for evaluation of the inter-brain synchrony, with theta–alpha hyper-brain subnetworks playing an essential role in the between-brain binding, and with alpha-frequency nodes serving a cleaving or pacemaker function in the HB-HFN (Müller and Lindenberger, 2014). Most interestingly, such a differentiation between WFC and CFC patterns (increasing WFC and decreasing CFC with advancing frequency) was also found in a superordinate physiological system with the respiratory, cardiac, and vocalizing subsystems in a choir in song, also including the motor subsystem of the choir conductor (Müller et al., 2018a). It can therefore be assumed that such system behaviors (with increasing WFC and decreasing CFC in relation to increasing frequency) are characteristic of biological systems and of organismic, but also social organizations. This is apparently due to the fact that WFC typically emphasizes the local features of complex systems, which operate relatively quickly and utilize faster frequencies for this purpose. In contrast, CFC tends to focus on the integrative capabilities of the system, which are better suited to slower or lower frequencies. These coordination dynamics are assumed to function as a superordinate system, or superorganism, based on the principles of self-organization and circular causality with upward and downward causation, which are emergent properties of the system (Müller et al., 2018a, 2019a; Delius and Müller, 2023).

Examining the dynamics within the music sequences showed that the variability in the network topology determined by the SD differs in the four guitarists and also varies with the electrode sites, oscillation frequency, and music sequences in the two pieces of music. Most interestingly, the SD decreased with higher frequency for all topology measures. This suggests that the low-frequency nodes, which exhibit most variability, may have adaptive capabilities to adequately adjust the system or HB-HFN to the changes that occur during guitar playing. Recent literature suggests that brain signal variability (i.e., transient temporal fluctuations in brain signal) can capture complex interactions between neuronal structures and cell assemblies and provide important information about network dynamics and brain states as well as cognitive performance and mental activity (McIntosh et al., 2008, 2014; Deco et al., 2011; Garrett et al., 2011, 2015; Sleimen-Malkoun et al., 2015). It has also been shown that the network structure and connectivity dynamics are non-stationary and reveal rich dynamic patterns, characterized by rapid transitions switching between a few discrete functional connectivity states (Betzel et al., 2012, 2016; Hansen et al., 2015; Shen et al., 2015). In addition, analysis of the temporal variability of HFN structure has revealed specific NTD, i.e., temporal changes of different GTA measures such as strength, *CC*, *CPL*, and local and global efficiency determined for HFNs in different time windows (Müller et al., 2016, 2019b). Furthermore, the variability of these NTD metrics, as measured by the SD over time, was found to correlate positively with perceptual speed, suggesting that a more variable NTD increases performance on cognitive or at least perceptual speed functions and improves the adaptability of the system or individual (Müller et al., 2019b). Thus, the high variability or adaptability of low-frequency nodes in the HB-HFN is accompanied by the integrative properties of these nodes as indicated by the shorter *CPL*.

It has been suggested that the real-time exchange of information between musicians that the ensemble needs to maintain coordination and achieve its artistic goals is determined by the social dynamics and constraints related to the musical material and instruments (Keller, 2014, 2023; Bishop, 2018; Bishop et al., 2021; Bishop and Keller, 2022). As mentioned above, the relationships between brains and instruments provide important evidence that inter-brain or hyper-brain synchrony has a specific relationship to the behavior of musicians (Müller and Lindenberger, 2023). Here we showed that different NTD measures exhibit linear and/or causal relationships with guitar sounds that vary in amplitude and frequency characteristics, with guitar sounds having a stronger influence on brain NTD than vice versa. These guitar-to-brain connections were also found for intra- and inter-brain strengths, especially during Libertango, but there were also connections going from brain to guitar, especially during Comme un tango. We also showed that high intra-brain strengths in a guitarist are strongly related to the inter-brain connectivity strengths from this guitarist to all others. Moreover, this relationship, when examined by multivariate GC, is mostly unidirectional and reaches from inter-brain to intra-brain strength, and may involve not only the same but also other guitarists. These influences from inter-brain to intra-brain strength presumably indicate that inter-brain synchrony can affect neural processes within the brains to achieve a stronger coordination of playing. In our previous study, we showed that these relationships between brains and instruments concern not only the guitarists’ but also the audience members’ brains during a concert (Müller and Lindenberger, 2023). In general, it can be concluded that the network topology of brains and the dynamical structure of guitar sounds are in permanent interplay and exhibit specific guitar–guitar, guitar–brain, and brain–brain bi- and unidirectional associations, indicating multilevel dynamics with upward and downward causation at all levels of dynamic organization.

We investigated the effect of edge or link removal in the entire HB-HFN or in its part concerning one guitarist’s brain and examined how this removal would change the network topology within the whole HB-HFN and in individual guitarists’ brains. We showed that the HB-HFN of the guitarist quartet is relatively robust against the loss of connections, especially when the strongest connections are preserved and especially when the loss of connections only affects one guitarist’s brain. When the edge removal or the loss of connections is manipulated in one guitarist’s brain, the topology measures (*CC* and *CPL*) change significantly only when the random and especially the strongest connections were lost, while the weakest connections mostly have no significant effect on the network topology. This indicates that the strongest connections play an essential role in the network topology and the loss of these connections may have detrimental consequences for topology and functioning. As mentioned above, network robustness is the ability of a network to maintain its integrity and functionality after the removal of nodes or edges, and is a prominent feature of most biological systems and social groups (Barabási and Pósfai, 2016; Liu et al., 2020; Bellingeri et al., 2020b). The fact that removing nodes according to weighted rank produces the highest damage in real-world complex networks is well known (Bellingeri and Cassi, 2018; Bellingeri et al., 2019, 2020a). Moreover, it has been found that the robustness of real-world complex networks against link removal is negatively correlated with link weights heterogeneity and that a small fraction of strong links removal can rapidly decrease the efficiency and the total flow in these systems (Bellingeri et al., 2019). All this indicates that removal of the strongest connections affects the functionality of a network not only because they are so strong, but mainly because the strongest connections are highly relevant in terms of network structure and its topology. In HB-HFN of the guitarist quartet, the low-frequency nodes have the strongest connections and play an important role in the functioning of the network, which is mainly integrated by these connections or nodes. If one would imagine a situation in which the quartet is disturbed in its functionality (e.g., by any technical disturbances or other external circumstances), these low-frequency connections between the quartet participants would probably be the first to be disrupted, and the network could become disorganized or disintegrated. On the other hand, it has recently been shown that the coupling between the brains of pianists can increase during a disturbance, presumably as an adaptive compensatory effect of inter-brain synchronization (Lender et al., 2023). This indicates that our simulations of link removal are very important to understand how networks react, or can react, to such perturbations, but they are not sufficient to draw conclusions about living organisms or intact social groups and interactions, as different adaptive compensatory mechanisms can counteract the perturbations in such networks. Further studies are needed to better understand such processes and phenomena.

### Limitations

The present study has limitations and leaves room for questions that should be addressed in future research. First, we considered the whole HB-HFN as a supra-adjacency matrix and computed the GTA measures for each individual node with respect to the whole network. The individual layers and the connections between them were only captured using WFC (as coupling within the layers) and CFC (as coupling between the layers) strengths. However, the multilayer structure could be assessed in an even more differentiated way. To do so, the GTA tools must be adapted or other tools must be used to differentiate between the layers. Furthermore, we only used three GTA measures (i.e., *S*, *CC*, and *CPL*). Other GTA measures could be helpful to capture other properties of these complex networks, such as assortativity, betweenness or closeness centrality, local and global efficiency, etc. Second, we analyzed the properties of the hyper-brain network within a single quartet. However, we observed consistent patterns of HB-HFN connectivity and network organization across different music sequences in two distinct pieces of music. While this may enhance the generalizability of the results, further research in this direction is warranted. Third, robustness of a complex network is an important property that has not yet been investigated in relation to hyper-brain networks. It may be useful to investigate such networks in a social interaction under perturbation conditions (cf. Lender et al., 2023) in order to verify and further develop the simulation assumptions. Furthermore, we investigated the removal of links, but removal of nodes would broaden the perspective of robustness, allowing specific nodes and their role in the network to be investigated. Therefore, further sophisticated research is needed to shed light on the neural mechanisms of social interaction and interpersonal action coordination behavior.

## Conclusion

Our results extend previous work on the reach of network interactions during interpersonal action coordination when playing the guitar in a quartet and highlight the way in which WFC and CFC, representing within- and between-layer communications in a complex multilayer HB-HFN, integrates different levels of network interaction with regard to its topology and functioning. We demonstrate linear and causal relationships between different characteristics of guitar sounds and GTA properties. We conclude that playing the guitar in a quartet is a dynamic process requiring tight interpersonal action coordination that is characterized by coupled brains and specific network topology dynamics, with high robustness of both network elements and underlying network structure. These findings align with studies investigating neural markers of interpersonal action coordination, particularly in the context of inter- or hyper-brain network activity (Sänger et al., 2012; Müller et al., 2013, 2018b; Müller and Lindenberger, 2014, 2019, 2023) and sensorimotor coupling in music and ensemble playing (Janata et al., 2012; Keller et al., 2014; van der Steen et al., 2015; Gallotti et al., 2017; Jacoby et al., 2021; Laroche et al., 2022). It is assumed that these coordination dynamics function as a superordinate system, or superorganism, based on the principles of self-organization and circular causality, which are emergent properties of system behavior. Our methods provide a versatile toolkit for studying interpersonal action coordination across various social interactions and behaviors.

## Data availability statement

The data analyzed in this study is subject to the following licenses/restrictions: the dataset is intended for internal use only. Requests to access these datasets should be directed to vmueller@mpib-berlin.mpg.de.

## Ethics statement

The studies involving humans were approved by the Ethics Committee of Max Planck Institute for Human Development approved the study. The studies were conducted in accordance with the local legislation and institutional requirements. The participants provided their written informed consent to participate in this study.

## Author contributions

VM: Conceptualization, Investigation, Methodology, Project administration, Visualization, Writing – original draft, Writing – review & editing, Data curation, Supervision. UL: Writing – original draft, Writing – review & editing, Conceptualization, Supervision.

## Supplementary material

SUPPLEMENTARY Video 1 This video contains a 30-second sequence from the quartet’s performance of Libertango during the experiment.

Supplementary Video 2 This video showcases real-time HB-HFN changes as the quartet performs a 5-second music sequence from Libertango.
