# Supplementary material for: Hyper-brain hyper-frequency network topology dynamics when playing guitar in quartet
Source: Front Hum Neurosci. 2024 Jun 11;18:1416667. doi: 10.3389/fnhum.2024.1416667 (PMC11196789; doi:10.3389/fnhum.2024.1416667)
Supplement: Supplementary file 1 [file Data_Sheet_1.PDF]

Supplementary Material

Hyper-brain hyper-frequency network topology dynamics when playing guitar in quartet

Viktor Müller and Ulman Lindenberger

Supplementary Tables

Supplementary Table 1. Music 5-s sequences in Libertango and Comme un Tango

Music sequence	Description
	Libertango (Astor Piazolla)
Sequence 1	Guitarist D is playing, and guitarists A, B, and C are starting to play
Sequence 2	All guitarists are playing
Sequence 3	Guitarists A and D are consonant[1]
Sequence 4	Guitarist B is accompanied by guitarists A, C, and D
Sequence 5	Musical "interruption" by guitarist A, who has previously only been accompanying
Sequence 6	Guitarists A and B are consonant
Sequence 7	Retransition to main theme
Sequence 8	Guitarists C and D are consonant
Sequence 9	Guitarists B, C, and D are consonant
Sequence 10	All guitarists (A, B, C, and D) are consonant
	Comme un Tango (Patrick Roux)
Sequence 1	Guitarist B responds to the calling of the guitarist A
Sequence 2	Guitarist D is playing, and guitarists A, B, and C are starting to play
Sequence 3	Guitarists A and B are consonant
Sequence 4	All guitarists are playing
Sequence 5	Guitarist B responds to the calling of the guitarist A, and guitarist D responds to the calling of the guitarist B
Sequence 6	Guitarist B is accompanied by guitarists A, C, and D
Sequence 7	Guitarist C responds to the calling of the guitarist A
Sequence 8	Guitarist D responds to the calling of the guitarist A
Sequence 9	Guitarist D responds to the calling of the guitarist A
Sequence 10	Musical interruption by guitarist D, who has previously been accompanying
Sequence 11	Retransition to main theme
Sequence 12	Guitarist D responds to the calling of the guitarist B
Sequence 13	*Calmando* und *molto ritardando* for all the guitarists
Sequence 14	Guitarists B and D are consonant

[1]Consonant: Guitarists are playing consonant intervals or sounds fitting to each other and sounding harmonious.

Supplementary Table 2. ANOVA results for the coupling *Strength* across the different guitarists, frequencies, sites, and sequences for Libertango (MP1)

Factors	df	F-value	P-value	η^2
Guitarist	3	92.907	0.000	0.236
Frequency	8	9236.301	0.000	0.988
Site	2	7.258	0.001	0.016
Guitarist * Frequency	24	36.261	0.000	0.492
Guitarist * Site	6	2.739	0.012	0.018
Frequency * Site	16	16.818	0.000	0.230
Guitarist * Frequency * Site	48	6.463	0.000	0.256
Error	900			
Sequence	7.500	138.873	0.000	0.134
Sequence * Guitarist	22.499	16.026	0.000	0.051
Sequence * Frequency	59.997	16.769	0.000	0.130
Sequence * Site	14.999	3.287	0.000	0.007
Sequence * Guitarist * Frequency	179.990	18.681	0.000	0.333
Sequence * Guitarist * Site	44.998	3.546	0.000	0.023
Sequence * Frequency * Site	119.993	3.891	0.000	0.065
Sequence * Guitarist * Frequency * Site	359.980	4.335	0.000	0.188
Error (Sequence)	6749.634			

Supplementary Table 3. Scheffé test for post-hoc differences in *Strength* between different factor levels for the factors Guitarist, Frequency, Site and Sequence in Libertango (MP1)

Levels	M. Diff.	P-Value	Levels	M. Diff.	P-Value
Guitarist			Sequence		
A, B	1.398	0.0012	S1, S2	1.215	0.3615
A, C	-0.076	0.9973	S1, S3	1.979	0.0019
A, D	-4.059	<0.0001	S1, S4	3.207	<0.0001
B, C	-1.475	0.0005	S1, S5	3.818	<0.0001
B, D	-5.458	<0.0001	S1, S6	8.634	<0.0001
C, D	-3.983	<0.0001	S1, S7	9.344	<0.0001
Frequency			S1, S8	3.033	<0.0001
f1, f2	-38.332	<0.0001	S1, S9	4.249	<0.0001
f1, f3	-29.088	<0.0001	S1, S10	-.739	0.9326
f1, f4	14.261	<0.0001	S2, S3	.764	0.9175
f1, f5	6.905	<0.0001	S2, S4	1.992	0.0017
f1, f6	73.086	<0.0001	S2, S5	2.603	<0.0001
f1, f7	26.155	<0.0001	S2, S6	7.419	<0.0001
f1, f8	49.542	<0.0001	S2, S7	8.129	<0.0001
f1, f9	42.078	<0.0001	S2, S8	1.819	0.0086
f2, f3	9.244	<0.0001	S2, S9	3.034	<0.0001
f2, f4	52.593	<0.0001	S2, S10	-1.954	0.0025
f2, f5	45.236	<0.0001	S3, S4	1.228	0.3442
f2, f6	111.417	<0.0001	S3, S5	1.839	0.0072
f2, f7	64.487	<0.0001	S3, S6	6.654	<0.0001
f2, f8	87.874	<0.0001	S3, S7	7.365	<0.0001
f2, f9	80.409	<0.0001	S3, S8	1.054	0.5927
f3, f4	43.349	<0.0001	S3, S9	2.270	<0.0001
f3, f5	35.992	<0.0001	S3, S10	-2.719	<0.0001
f3, f6	102.173	<0.0001	S4, S5	.611	0.9810
f3, f7	55.243	<0.0001	S4, S6	5.426	<0.0001
f3, f8	78.630	<0.0001	S4, S7	6.137	<0.0001
f3, f9	71.165	<0.0001	S4, S8	-.174	>.9999
f4, f5	-7.357	<0.0001	S4, S9	1.042	0.6106
f4, f6	58.824	<0.0001	S4, S10	-3.947	<0.0001
f4, f7	11.894	<0.0001	S5, S6	4.815	<0.0001
f4, f8	35.281	<0.0001	S5, S7	5.526	<0.0001
f4, f9	27.816	<0.0001	S5, S8	-.785	0.9034
f5, f6	66.181	<0.0001	S5, S9	.431	0.9987
f5, f7	19.250	<0.0001	S5, S10	-4.558	<0.0001
f5, f8	42.637	<0.0001	S6, S7	.711	0.9475
f5, f9	35.173	<0.0001	S6, S8	-5.600	<0.0001
f6, f7	-46.931	<0.0001	S6, S9	-4.385	<0.0001
f6, f8	-23.544	<0.0001	S6, S10	-9.373	<0.0001
f6, f9	-31.008	<0.0001	S7, S8	-6.311	<0.0001
f7, f8	23.387	<0.0001	S7, S9	-5.095	<0.0001
f7, f9	15.923	<0.0001	S7, S10	-10.084	<0.0001
f8, f9	-7.464	<0.0001	S8, S9	1.216	0.3606
Site			S8, S10	-3.773	<0.0001
F, C	-1.057	0.0047	S9, S10	-4.989	<0.0001
F, P	0.052	0.9837			
C, P	1.109	0.0022			

Supplementary Table 4. ANOVA results for the coupling *Strength* across the different guitarists, frequencies, sites, and sequences for Comme un Tango (MP2)

Factors	df	F-value	P-value	η^2
Guitarist	3	66.576	0.000	0.182
Frequency	8	14178.503	0.000	0.992
Site	2	14.109	0.000	0.030
Guitarist * Frequency	24	48.203	0.000	0.562
Guitarist * Site	6	11.749	0.000	0.073
Frequency * Site	16	9.630	0.000	0.146
Guitarist * Frequency * Site	48	4.452	0.000	0.192
Error	900			
Sequence	11.951	66.040	0.000	0.068
Sequence * Guitarist	35.852	17.363	0.000	0.055
Sequence * Frequency	95.606	11.788	0.000	0.095
Sequence * Site	23.901	3.919	0.000	0.009
Sequence * Guitarist * Frequency	286.817	18.640	0.000	0.332
Sequence * Guitarist * Site	71.704	3.817	0.000	0.025
Sequence * Frequency * Site	191.212	5.613	0.000	0.091
Sequence * Guitarist * Frequency * Site	573.635	4.529	0.000	0.195
Error (Sequence)	10755.651			

Supplementary Table 5. Scheffé test for post-hoc differences in *Strength* between different factor levels for the factors Guitarist, Frequency, Site and Sequence in Comme un tango (MP2)

Levels	M. Diff.	P-Value	Levels	M. Diff.	P-Value	Levels	M. Diff.	P-Value
Guitarist			Sequence					
A, B	1.746	<0.0001	S1, S2	0.856	0.9832	S5, S7	-1.352	0.5749
A, C	-0.668	0.1371	S1, S3	0.247	>0.999	S5, S8	-2.644	<0.0001
A, D	-2.209	<0.0001	S1, S4	1.572	0.2796	S5, S9	0.635	0.999
B, C	-2.414	<0.0001	S1, S5	6.516	<0.0001	S5, S10	-1.634	0.2132
B, D	-3.955	<0.0001	S1, S6	2.950	<0.0001	S5, S11	-.546	>0.999
C, D	-1.541	<0.0001	S1, S7	5.164	<0.0001	S5, S12	-3.334	<0.0001
Frequency			S1, S8	3.872	<0.0001	S5, S13	-.926	0.966
f1, f2	-25.957	<0.0001	S1, S9	7.150	<0.0001	S5, S14	-1.798	0.0901
f1, f3	-17.201	<0.0001	S1, S10	4.882	<0.0001	S6, S7	2.214	0.0038
f1, f4	24.360	<0.0001	S1, S11	5.969	<0.0001	S6, S8	0.922	0.9674
f1, f5	14.571	<0.0001	S1, S12	3.182	<0.0001	S6, S9	4.200	<0.0001
f1, f6	83.062	<0.0001	S1, S13	5.590	<0.0001	S6, S10	1.932	0.0378
f1, f7	37.073	<0.0001	S1, S14	4.718	<0.0001	S6, S11	3.019	<0.0001
f1, f8	59.416	<0.0001	S2, S3	-0.609	0.9995	S6, S12	.232	>0.999
f1, f9	54.286	<0.0001	S2, S4	0.716	0.9971	S6, S13	2.639	<0.0001
f2, f3	8.756	<0.0001	S2, S5	5.660	<0.0001	S6, S14	1.768	0.1069
f2, f4	50.317	<0.0001	S2, S6	2.094	0.0109	S7, S8	-1.292	0.6570
f2, f5	40.528	<0.0001	S2, S7	4.308	<0.0001	S7, S9	1.986	0.0255
f2, f6	109.019	<0.0001	S2, S8	3.016	<0.0001	S7, S10	-0.283	>0.999
f2, f7	63.030	<0.0001	S2, S9	6.294	<0.0001	S7, S11	0.805	0.9906
f2, f8	85.373	<0.0001	S2, S10	4.025	<0.0001	S7, S12	-1.983	0.0262
f2, f9	80.243	<0.0001	S2, S11	5.113	<0.0001	S7, S13	.425	>0.999
f3, f4	41.560	<0.0001	S2, S12	2.325	0.0013	S7, S14	-0.446	>0.999
f3, f5	31.772	<0.0001	S2, S13	4.733	<0.0001	S8, S9	3.278	<0.0001
f3, f6	100.262	<0.0001	S2, S14	3.862	<0.0001	S8, S10	1.010	0.9313
f3, f7	54.274	<0.0001	S3, S4	1.325	0.6126	S8, S11	2.097	0.0106
f3, f8	76.617	<0.0001	S3, S5	6.268	<0.0001	S8, S12	-0.690	0.998
f3, f9	71.487	<0.0001	S3, S6	2.703	<0.0001	S8, S13	1.717	0.1411
f4, f5	-9.789	<0.0001	S3, S7	4.917	<0.0001	S8, S14	0.846	0.985
f4, f6	58.702	<0.0001	S3, S8	3.625	<0.0001	S9, S10	-2.269	0.0023
f4, f7	12.713	<0.0001	S3, S9	6.903	<0.0001	S9, S11	-1.181	0.793
f4, f8	35.056	<0.0001	S3, S10	4.634	<0.0001	S9, S12	-3.969	<0.0001
f4, f9	29.926	<0.0001	S3, S11	5.722	<0.0001	S9, S13	-1.561	0.2925
f5, f6	68.491	<0.0001	S3, S12	2.934	<0.0001	S9, S14	-2.432	0.0004
f5, f7	22.502	<0.0001	S3, S13	5.342	<0.0001	S10, S11	1.088	0.8802
f5, f8	44.845	<0.0001	S3, S14	4.471	<0.0001	S10, S12	-1.700	0.1545
f5, f9	39.715	<0.0001	S4, S5	4.944	<0.0001	S10, S13	0.708	0.9974
f6, f7	-45.989	<0.0001	S4, S6	1.378	0.5372	S10, S14	-.164	>0.999
f6, f8	-23.646	<0.0001	S4, S7	3.592	<0.0001	S11, S12	-2.788	<0.0001
f6, f9	-28.776	<0.0001	S4, S8	2.300	0.0017	S11, S13	-0.380	>0.999
f7, f8	22.343	<0.0001	S4, S9	5.578	<0.0001	S11, S14	-1.251	0.7106
f7, f9	17.213	<0.0001	S4, S10	3.310	<0.0001	S12, S13	2.408	0.0005
f8, f9	-5.130	<0.0001	S4, S11	4.397	<0.0001	S12, S14	1.537	0.3219
Site			S4, S12	1.610	0.2380	S13, S14	-0.871	0.9802
F, C	-1.349	<0.0001	S4, S13	4.018	<0.0001			
F, P	-0.818	0.0021	S4, S14	3.146	<0.0001			
C, P	0.532	0.1178	S5, S6	-3.566	<0.0001			

Supplementary Table 6. ANOVA results for the *Clustering Coefficient (CC)* across the different guitarists, frequencies, sites, and sequences for Libertango (MP1)

Factors	df	F-value	P-value	η^2
Guitarist	3,900	480.484	0.000	0.616
Frequency	8,900	1514.831	0.000	0.931
Site	2,900	34.150	0.000	0.071
Guitarist * Frequency	24,900	11.142	0.000	0.229
Guitarist * Site	6,900	17.067	0.000	0.102
Frequency * Site	16,900	5.862	0.000	0.094
Guitarist * Frequency * Site	48,900	2.586	0.000	0.121
Error				
Sequence	7.724	1436.083	0.000	0.615
Sequence * Guitarist	23.171	118.219	0.000	0.283
Sequence * Frequency	61.790	42.121	0.000	0.272
Sequence * Site	15.447	7.527	0.000	0.016
Sequence * Guitarist * Frequency	185.370	19.361	0.000	0.340
Sequence * Guitarist * Site	46.342	5.687	0.000	0.037
Sequence * Frequency * Site	123.580	3.424	0.000	0.057
Sequence * Guitarist * Frequency * Site	370.740	3.656	0.000	0.163
Error (Sequence)	6951.368			

Supplementary Table 7. Scheffé test for post-hoc differences in *Clustering Coefficient (CC)* between different factor levels for the factors Guitarist, Frequency, Site and Sequence in Libertango (MP1)

Levels	M. Diff.	P-Value	Levels	M. Diff.	P-Value
Guitarist			Sequence		
A, B	-0.002	<0.0001	S1, S2	0.001	<0.0001
A, C	-0.001	<0.0001	S1, S3	0.003	<0.0001
A, D	-0.004	<0.0001	S1, S4	0.003	<0.0001
B, C	0.0004	0.0078	S1, S5	0.003	<0.0001
B, D	-0.003	<0.0001	S1, S6	0.005	<0.0001
C, D	-0.003	<0.0001	S1, S7	0.005	<0.0001
Frequency			S1, S8	0.002	<0.0001
f1, f2	0.003	<0.0001	S1, S9	0.003	<0.0001
f1, f3	-0.0004	0.667	S1, S10	0.00024	0.0664
f1, f4	-0.005	<0.0001	S2, S3	0.002	<0.0001
f1, f5	-0.003	<0.0001	S2, S4	0.002	<0.0001
f1, f6	-0.011	<0.0001	S2, S5	0.002	<0.0001
f1, f7	-0.006	<0.0001	S2, S6	0.003	<0.0001
f1, f8	-0.011	<0.0001	S2, S7	0.004	<0.0001
f1, f9	-0.002	<0.0001	S2, S8	0.001	<0.0001
f2, f3	-0.003	<0.0001	S2, S9	0.001	<0.0001
f2, f4	-0.008	<0.0001	S2, S10	-0.001	<0.0001
f2, f5	-0.006	<0.0001	S3, S4	0.000003	0.9999
f2, f6	-0.014	<0.0001	S3, S5	-0.00001	0.999
f2, f7	-0.009	<0.0001	S3, S6	0.002	<0.0001
f2, f8	-0.014	<0.0001	S3, S7	0.002	<0.0001
f2, f9	-0.004	<0.0001	S3, S8	-0.001	<0.0001
f3, f4	-0.005	<0.0001	S3, S9	-0.0004	<0.0001
f3, f5	-0.003	<0.0001	S3, S10	-0.003	<0.0001
f3, f6	-0.010	<0.0001	S4, S5	-0.00001	0.9989
f3, f7	-0.006	<0.0001	S4, S6	0.002	<0.0001
f3, f8	-0.011	<0.0001	S4, S7	0.002	<0.0001
f3, f9	-0.001	<0.0001	S4, S8	-0.001	<0.0001
f4, f5	0.002	<0.0001	S4, S9	-0.0004	<0.0001
f4, f6	-0.006	<0.0001	S4, S10	-0.003	<0.0001
f4, f7	-0.001	<0.0001	S5, S6	0.002	<0.0001
f4, f8	-0.006	<0.0001	S5, S7	0.002	<0.0001
f4, f9	0.003	<0.0001	S5, S8	-0.001	<0.0001
f5, f6	-0.007	<0.0001	S5, S9	-0.0003	0.0003
f5, f7	-0.003	<0.0001	S5, S10	-0.003	<0.0001
f5, f8	-0.008	<0.0001	S6, S7	0.0003	0.0005
f5, f9	0.002	<0.0001	S6, S8	-0.003	<0.0001
f6, f7	0.005	<0.0001	S6, S9	-0.002	<0.0001
f6, f8	-0.0004	0.7212	S6, S10	-0.004	<0.0001
f6, f9	0.009	<0.0001	S7, S8	-0.003	<0.0001
f7, f8	-0.005	<0.0001	S7, S9	-0.002	<0.0001
f7, f9	0.005	<0.0001	S7, S10	-0.005	<0.0001
f8, f9	0.010	<0.0001	S8, S9	0.001	<0.0001
Site			S8, S10	-0.001	<0.0001
F, C	-0.001	<0.0001	S9, S10	-0.002	<0.0001
F, P	-0.001	<0.0001			
C, P	-0.00023	0.084			

Supplementary Table 8. ANOVA results for the *Clustering Coefficient (CC)* across the different guitarists, frequencies, sites, and sequences for Comme un Tango (MP2)

Factors	df	F-value	P-value	η^2
Guitarist	3	179.646	0.000	0.375
Frequency	8	1249.996	0.000	0.917
Site	2	98.873	0.000	0.180
Guitarist * Frequency	24	3.872	0.000	0.094
Guitarist * Site	6	27.567	0.000	0.155
Frequency * Site	16	5.987	0.000	0.096
Guitarist * Frequency * Site	48	1.772	0.001	0.086
Error				
Sequence	11.861	1403.206	0.000	0.609
Sequence * Guitarist	35.584	101.885	0.000	0.254
Sequence * Frequency	94.890	41.611	0.000	0.270
Sequence * Site	23.722	3.146	0.000	0.007
Sequence * Guitarist * Frequency	284.669	18.384	0.000	0.329
Sequence * Guitarist * Site	71.167	7.260	0.000	0.046
Sequence * Frequency * Site	189.779	4.647	0.000	0.076
Sequence * Guitarist * Frequency * Site	569.338	3.523	0.000	0.158
Error (Sequence)	10675.090			

Supplementary Table 9. Scheffé test for post-hoc differences in *Clustering Coefficient (CC)* between different factor levels for the factors Guitarist, Frequency, Site and Sequence in Comme un tango (MP2)

Levels	M. Diff.	P-Value	Levels	M. Diff.	P-Value	Levels	M. Diff.	P-Value
Guitarist			Sequence					
A, B	-0.0004	0.0262	S1, S2	0.001	<0.0001	S5, S7	0.001	<0.0001
A, C	-0.001	<0.0001	S1, S3	0.001	<0.0001	S5, S8	0.001	<0.0001
A, D	-0.002	<0.0001	S1, S4	0.002	<0.0001	S5, S9	0.002	<0.0001
B, C	0.001	<0.0001	S1, S5	0.003	<0.0001	S5, S10	0.002	<0.0001
B, D	-0.002	<0.0001	S1, S6	0.002	<0.0001	S5, S11	0.001	<0.0001
C, D	-0.003	<0.0001	S1, S7	0.005	<0.0001	S5, S12	-0.001	<0.0001
Frequency			S1, S8	0.004	<0.0001	S5, S13	0.001	<0.0001
f1, f2	0.002	<0.0001	S1, S9	0.005	<0.0001	S5, S14	0.001	<0.0001
f1, f3	-0.002	<0.0001	S1, S10	0.005	<0.0001	S6, S7	0.002	<0.0001
f1, f4	-0.006	<0.0001	S1, S11	0.004	<0.0001	S6, S8	0.002	<0.0001
f1, f5	-0.005	<0.0001	S1, S12	0.002	<0.0001	S6, S9	0.003	<0.0001
f1, f6	-0.012	<0.0001	S1, S13	0.005	<0.0001	S6, S10	0.002	<0.0001
f1, f7	-0.007	<0.0001	S1, S14	0.005	<0.0001	S6, S11	0.002	<0.0001
f1, f8	-0.013	<0.0001	S2, S3	0.001	<0.0001	S6, S12	-0.00006	>0.999
f1, f9	-0.002	<0.0001	S2, S4	0.001	<0.0001	S6, S13	0.002	<0.0001
f2, f3	-0.004	<0.0001	S2, S5	0.003	<0.0001	S6, S14	0.002	<0.0001
f2, f4	-0.008	<0.0001	S2, S6	0.002	<0.0001	S7, S8	-0.0003	0.0429
f2, f5	-0.007	<0.0001	S2, S7	0.004	<0.0001	S7, S9	0.001	<0.0001
f2, f6	-0.014	<0.0001	S2, S8	0.004	<0.0001	S7, S10	0.0003	0.1583
f2, f7	-0.008	<0.0001	S2, S9	0.005	<0.0001	S7, S11	-0.0004	<0.0001
f2, f8	-0.014	<0.0001	S2, S10	0.004	<0.0001	S7, S12	-0.002	<0.0001
f2, f9	-0.004	<0.0001	S2, S11	0.003	<0.0001	S7, S13	0.00001	>0.999
f3, f4	-0.004	<0.0001	S2, S12	0.002	<0.0001	S7, S14	0	>0.999
f3, f5	-0.003	<0.0001	S2, S13	0.004	<0.0001	S8, S9	0.001	<0.0001
f3, f6	-0.010	<0.0001	S2, S14	0.004	<0.0001	S8, S10	0.001	<0.0001
f3, f7	-0.005	<0.0001	S3, S4	0.001	<0.0001	S8, S11	-0.0001	0.9743
f3, f8	-0.011	<0.0001	S3, S5	0.002	<0.0001	S8, S12	-0.002	<0.0001
f3, f9	-0.0002	0.997	S3, S6	0.001	<0.0001	S8, S13	0.0003	0.0229
f4, f5	0.001	<0.0001	S3, S7	0.003	<0.0001	S8, S14	0.0003	0.0411
f4, f6	-0.006	<0.0001	S3, S8	0.003	<0.0001	S9, S10	-0.001	<0.0001
f4, f7	-0.001	0.0625	S3, S9	0.004	<0.0001	S9, S11	-0.001	<0.0001
f4, f8	-0.007	<0.0001	S3, S10	0.004	<0.0001	S9, S12	-0.003	<0.0001
f4, f9	0.004	<0.0001	S3, S11	0.003	<0.0001	S9, S13	-0.001	<0.0001
f5, f6	-0.007	<0.0001	S3, S12	0.001	<0.0001	S9, S14	-0.001	<0.0001
f5, f7	-0.002	<0.0001	S3, S13	0.003	<0.0001	S10, S11	-0.001	<0.0001
f5, f8	-0.008	<0.0001	S3, S14	0.003	<0.0001	S10, S12	-0.003	<0.0001
f5, f9	0.003	<0.0001	S4, S5	0.001	<0.0001	S10, S13	-0.00025	0.2397
f6, f7	0.005	<0.0001	S4, S6	0.001	<0.0001	S10, S14	-0.0003	0.1634
f6, f8	-0.001	0.3035	S4, S7	0.003	<0.0001	S11, S12	-0.002	<0.0001
f6, f9	0.010	<0.0001	S4, S8	0.002	<0.0001	S11, S13	0.0005	<0.0001
f7, f8	-0.006	<0.0001	S4, S9	0.003	<0.0001	S11, S14	0.0004	<0.0001
f7, f9	0.005	<0.0001	S4, S10	0.003	<0.0001	S12, S13	0.002	<0.0001
f8, f9	0.011	<0.0001	S4, S11	0.002	<0.0001	S12, S14	0.002	<0.0001
Site			S4, S12	0.0004	<0.0001	S13, S14	-0.00001	>0.999
F, C	-0.001	<0.0001	S4, S13	0.003	<0.0001			
F, P	-0.001	<0.0001	S4, S14	0.003	<0.0001			
C, P	-0.0004	0.0002	S5, S6	-0.001	<0.0001			

Supplementary Table 10. ANOVA results for the *Characteristic Path Length (CPL)* across the different guitarists, frequencies, sites, and sequences for Libertango (MP1)

Factors	df	F-value	P-value	η^2
Guitarist	3	120.757	0.000	0.287
Frequency	8	25362.319	0.000	0.996
Site	2	6.803	0.001	0.015
Guitarist * Frequency	24	45.572	0.000	0.549
Guitarist * Site	6	4.672	0.000	0.030
Frequency * Site	16	11.572	0.000	0.171
Guitarist * Frequency * Site	48	5.899	0.000	0.239
Error				
Sequence	7.534	279.914	0.000	0.237
Sequence * Guitarist	22.603	14.337	0.000	0.046
Sequence * Frequency	60.273	14.845	0.000	0.117
Sequence * Site	15.068	2.718	0.000	0.006
Sequence * Guitarist * Frequency	180.820	17.235	0.000	0.315
Sequence * Guitarist * Site	45.205	3.042	0.000	0.020
Sequence * Frequency * Site	120.547	3.874	0.000	0.064
Sequence * Guitarist * Frequency * Site	361.641	4.099	0.000	0.179
Error (Sequence)	6780.767			

Supplementary Table 11. Scheffé test for post-hoc differences in *Characteristic Path Length (CPL)* between different factor levels for the factors Guitarist, Frequency, Site and Sequence in Libertango (MP1)

Levels	M. Diff.	P-Value	Levels	M. Diff.	P-Value
Guitarist			Sequence		
A, B	-0.016	<0.0001	S1, S2	-0.005	0.7595
A, C	-0.004	0.1926	S1, S3	-0.009	0.0126
A, D	0.018	<0.0001	S1, S4	-0.018	<0.0001
B, C	0.012	<0.0001	S1, S5	-0.024	<0.0001
B, D	0.034	<0.0001	S1, S6	-0.059	<0.0001
C, D	0.022	<0.0001	S1, S7	-0.065	<0.0001
Frequency			S1, S8	-0.011	0.0005
f1, f2	0.124	<0.0001	S1, S9	-0.021	<0.0001
f1, f3	-0.007	0.4703	S1, S10	0.015	<0.0001
f1, f4	-0.233	<0.0001	S2, S3	-0.004	0.8566
f1, f5	-0.289	<0.0001	S2, S4	-0.013	<0.0001
f1, f6	-0.579	<0.0001	S2, S5	-0.019	<0.0001
f1, f7	-0.455	<0.0001	S2, S6	-0.054	<0.0001
f1, f8	-0.611	<0.0001	S2, S7	-0.061	<0.0001
f1, f9	-0.749	<0.0001	S2, S8	-0.006	0.4012
f2, f3	-0.131	<0.0001	S2, S9	-0.016	<0.0001
f2, f4	-0.357	<0.0001	S2, S10	0.019	<0.0001
f2, f5	-0.413	<0.0001	S3, S4	-0.009	0.0400
f2, f6	-0.703	<0.0001	S3, S5	-0.014	<0.0001
f2, f7	-0.579	<0.0001	S3, S6	-0.050	<0.0001
f2, f8	-0.734	<0.0001	S3, S7	-0.056	<0.0001
f2, f9	-0.873	<0.0001	S3, S8	-0.002	0.9998
f3, f4	-0.226	<0.0001	S3, S9	-0.012	0.0002
f3, f5	-0.282	<0.0001	S3, S10	0.024	<0.0001
f3, f6	-0.572	<0.0001	S4, S5	-0.006	0.5536
f3, f7	-0.448	<0.0001	S4, S6	-0.041	<0.0001
f3, f8	-0.603	<0.0001	S4, S7	-0.047	<0.0001
f3, f9	-0.741	<0.0001	S4, S8	0.007	0.2798
f4, f5	-0.056	<0.0001	S4, S9	-0.003	0.9868
f4, f6	-0.346	<0.0001	S4, S10	0.033	<0.0001
f4, f7	-0.222	<0.0001	S5, S6	-0.035	<0.0001
f4, f8	-0.377	<0.0001	S5, S7	-0.042	<0.0001
f4, f9	-0.515	<0.0001	S5, S8	0.013	<0.0001
f5, f6	-0.290	<0.0001	S5, S9	0.003	0.9957
f5, f7	-0.166	<0.0001	S5, S10	0.038	<0.0001
f5, f8	-0.321	<0.0001	S6, S7	-0.006	0.3817
f5, f9	-0.459	<0.0001	S6, S8	0.048	<0.0001
f6, f7	0.124	<0.0001	S6, S9	0.038	<0.0001
f6, f8	-0.031	<0.0001	S6, S10	0.074	<0.0001
f6, f9	-0.169	<0.0001	S7, S8	0.054	<0.0001
f7, f8	-0.155	<0.0001	S7, S9	0.044	<0.0001
f7, f9	-0.294	<0.0001	S7, S10	0.080	<0.0001
f8, f9	-0.138	<0.0001	S8, S9	-0.010	0.0060
Site			S8, S10	0.026	<0.0001
F, C	0.006	0.0027	S9, S10	0.036	<0.0001
F, P	0.01	0.9019			
C, P	-0.005	0.0082			

Supplementary Table 12. ANOVA results for the *Characteristic Path Length (CPL)* across the different guitarists, frequencies, sites, and sequences for Comme un Tango (MP2)

Factors	df	F-value	P-value	η^2
Guitarist	3	102.523	0.000	0.255
Frequency	8	41610.572	0.000	0.997
Site	2	13.711	0.000	0.030
Guitarist * Frequency	24	47.621	0.000	0.559
Guitarist * Site	6	6.716	0.000	0.043
Frequency * Site	16	10.626	0.000	0.159
Guitarist * Frequency * Site	48	3.956	0.000	0.174
Error				
Sequence	12.225	121.917	0.000	0.119
Sequence * Guitarist	36.675	16.856	0.000	0.053
Sequence * Frequency	97.800	11.327	0.000	0.091
Sequence * Site	24.450	3.229	0.000	0.007
Sequence * Guitarist * Frequency	293.399	17.265	0.000	0.315
Sequence * Guitarist * Site	73.350	3.453	0.000	0.023
Sequence * Frequency * Site	195.600	4.833	0.000	0.079
Sequence * Guitarist * Frequency * Site	586.799	4.193	0.000	0.183
Error (Sequence)	11002.481			

Supplementary Table 13. Scheffé test for post-hoc differences in *Characteristic Path Length (CPL)* between different factor levels for the factors Guitarist, Frequency, Site and Sequence in Comme un tango (MP2)

Levels	M. Diff.	P-Value	Levels	M. Diff.	P-Value	Levels	M. Diff.	P-Value
Guitarist			Sequence					
A, B	-0.011	<0.0001	S1, S2	-0.011	<0.0001	S5, S7	0.010	0.0459
A, C	0.007	<0.0001	S1, S3	-0.002	>0.999	S5, S8	0.013	0.0001
A, D	0.013	<0.0001	S1, S4	-0.010	0.0381	S5, S9	-0.003	>0.999
B, C	0.018	<0.0001	S1, S5	-0.046	<0.0001	S5, S10	0.015	<0.0001
B, D	0.024	<0.0001	S1, S6	-0.026	<0.0001	S5, S11	-0.001	>0.999
C, D	0.007	<0.0001	S1, S7	-0.036	<0.0001	S5, S12	0.018	<0.0001
Frequency			S1, S8	-0.033	<0.0001	S5, S13	0.007	0.6015
f1, f2	0.065	<0.0001	S1, S9	-0.049	<0.0001	S5, S14	0.011	0.0038
f1, f3	-0.066	<0.0001	S1, S10	-0.031	<0.0001	S6, S7	-0.010	0.0282
f1, f4	-0.280	<0.0001	S1, S11	-0.046	<0.0001	S6, S8	-0.007	0.5821
f1, f5	-0.327	<0.0001	S1, S12	-0.028	<0.0001	S6, S9	-0.023	<0.0001
f1, f6	-0.626	<0.0001	S1, S13	-0.039	<0.0001	S6, S10	-0.005	0.9546
f1, f7	-0.512	<0.0001	S1, S14	-0.034	<0.0001	S6, S11	-0.020	<0.0001
f1, f8	-0.657	<0.0001	S2, S3	0.009	0.104	S6, S12	-0.002	>0.999
f1, f9	-0.810	<0.0001	S2, S4	0.001	>0.999	S6, S13	-0.013	0.0001
f2, f3	-0.130	<0.0001	S2, S5	-0.035	<0.0001	S6, S14	-0.008	0.1860
f2, f4	-0.344	<0.0001	S2, S6	-0.015	<0.0001	S7, S8	0.003	>0.999
f2, f5	-0.392	<0.0001	S2, S7	-0.025	<0.0001	S7, S9	-0.013	0.0002
f2, f6	-0.691	<0.0001	S2, S8	-0.022	<0.0001	S7, S10	0.005	0.929
f2, f7	-0.576	<0.0001	S2, S9	-0.038	<0.0001	S7, S11	-0.010	0.0182
f2, f8	-0.722	<0.0001	S2, S10	-0.020	<0.0001	S7, S12	0.008	0.2049
f2, f9	-0.875	<0.0001	S2, S11	-0.035	<0.0001	S7, S13	-0.003	>0.999
f3, f4	-0.214	<0.0001	S2, S12	-0.017	<0.0001	S7, S14	0.002	>0.999
f3, f5	-0.261	<0.0001	S2, S13	-0.028	<0.0001	S8, S9	-0.016	<0.0001
f3, f6	-0.560	<0.0001	S2, S14	-0.024	<0.0001	S8, S10	0.002	>0.999
f3, f7	-0.446	<0.0001	S3, S4	-0.008	0.268	S8, S11	-0.014	<0.0001
f3, f8	-0.592	<0.0001	S3, S5	-0.044	<0.0001	S8, S12	0.005	0.9276
f3, f9	-0.745	<0.0001	S3, S6	-0.024	<0.0001	S8, S13	-0.006	0.7749
f4, f5	-0.047	<0.0001	S3, S7	-0.034	<0.0001	S8, S14	-0.002	>0.999
f4, f6	-0.346	<0.0001	S3, S8	-0.031	<0.0001	S9, S10	0.018	<0.0001
f4, f7	-0.232	<0.0001	S3, S9	-0.047	<0.0001	S9, S11	0.002	>0.999
f4, f8	-0.378	<0.0001	S3, S10	-0.029	<0.0001	S9, S12	0.021	<0.0001
f4, f9	-0.531	<0.0001	S3, S11	-0.044	<0.0001	S9, S13	0.010	0.0335
f5, f6	-0.299	<0.0001	S3, S12	-0.026	<0.0001	S9, S14	0.014	<0.0001
f5, f7	-0.185	<0.0001	S3, S13	-0.037	<0.0001	S10, S11	-0.015	<0.0001
f5, f8	-0.330	<0.0001	S3, S14	-0.033	<0.0001	S10, S12	0.003	>0.999
f5, f9	-0.483	<0.0001	S4, S5	-0.036	<0.0001	S10, S13	-0.008	0.2649
f6, f7	0.114	<0.0001	S4, S6	-0.016	<0.0001	S10, S14	-0.004	0.9975
f6, f8	-0.031	<0.0001	S4, S7	-0.026	<0.0001	S11, S12	0.019	<0.0001
f6, f9	-0.184	<0.0001	S4, S8	-0.023	<0.0001	S11, S13	0.007	0.4235
f7, f8	-0.145	<0.0001	S4, S9	-0.039	<0.0001	S11, S14	0.012	0.0011
f7, f9	-0.298	<0.0001	S4, S10	-0.021	<0.0001	S12, S13	-0.011	0.0038
f8, f9	-0.153	<0.0001	S4, S11	-0.036	<0.0001	S12, S14	-0.007	0.6012
Site			S4, S12	-0.018	<0.0001	S13, S14	0.004	0.9782
F, C	0.006	<0.0001	S4, S13	-0.029	<0.0001			
F, P	0.005	0.0006	S4, S14	-0.025	<0.0001			
C, P	-0.002	0.3292	S5, S6	0.020	<0.0001			

Supplementary Table 14. ANOVA results for SD of the *Strength (S)* across the different guitarists, frequencies, sites, and sequences for Libertango (MP1)

Factors	df	F-value	P-value	η^2
Guitarist	3	7.029	0.000	0.023
Frequency	8	9957.388	0.000	0.989
Site	2	7.466	0.001	0.016
Guitarist * Frequency	24	8.926	0.000	0.192
Guitarist * Site	6	1.959	0.069	0.013
Frequency * Site	16	4.767	0.000	0.078
Guitarist * Frequency * Site	48	3.845	0.000	0.170
Error	900			
Sequence	7.879	7.430	0.000	0.008
Sequence * Guitarist	23.637	7.078	0.000	0.023
Sequence * Frequency	63.033	3.991	0.000	0.034
Sequence * Site	15.758	2.700	0.000	0.006
Sequence * Guitarist * Frequency	189.100	5.475	0.000	0.127
Sequence * Guitarist * Site	47.275	2.702	0.000	0.018
Sequence * Frequency * Site	126.067	2.074	0.000	0.036
Sequence * Guitarist * Frequency * Site	378.200	1.954	0.000	0.094
Error (Sequence)	7091.244			

Supplementary Table 15. Scheffé test for post-hoc differences in SD of the *Strength (S)* between different factor levels for the factors Guitarist, Frequency, Site and Sequence in Libertango (MP1)

Levels	M. Diff.	P-Value	Levels	M. Diff.	P-Value
Guitarist			Sequence		
A, B	-0.591	0.0084	S1, S2	0.576	0.8284
A, C	-0.569	0.0123	S1, S3	1.036	0.0597
A, D	-0.001	>0.999	S1, S4	0.505	0.9181
B, C	0.022	0.9995	S1, S5	0.775	0.4226
B, D	0.590	0.0085	S1, S6	0.749	0.4799
C, D	0.568	0.0125	S1, S7	0.913	0.1754
Frequency			S1, S8	0.412	0.9784
f1, f2	20.929	<0.0001	S1, S9	0.154	>0.9999
f1, f3	35.904	<0.0001	S1, S10	-0.644	0.7070
f1, f4	46.492	<0.0001	S2, S3	0.460	0.9548
f1, f5	47.677	<0.0001	S2, S4	-0.071	>0.9999
f1, f6	55.350	<0.0001	S2, S5	0.199	>0.9999
f1, f7	51.119	<0.0001	S2, S6	0.172	>0.9999
f1, f8	53.978	<0.0001	S2, S7	0.337	0.9950
f1, f9	51.426	<0.0001	S2, S8	-0.164	>0.9999
f2, f3	14.975	<0.0001	S2, S9	-0.423	0.9742
f2, f4	25.563	<0.0001	S2, S10	-1.220	0.0069
f2, f5	26.748	<0.0001	S3, S4	-0.531	0.8909
f2, f6	34.421	<0.0001	S3, S5	-0.261	0.9993
f2, f7	30.191	<0.0001	S3, S6	-0.287	0.9986
f2, f8	33.049	<0.0001	S3, S7	-0.122	>0.9999
f2, f9	30.497	<0.0001	S3, S8	-0.624	0.7459
f3, f4	10.588	<0.0001	S3, S9	-0.882	0.2206
f3, f5	11.772	<0.0001	S3, S10	-1.680	<0.0001
f3, f6	19.446	<0.0001	S4, S5	0.270	0.9991
f3, f7	15.215	<0.0001	S4, S6	0.243	0.9996
f3, f8	18.074	<0.0001	S4, S7	0.408	0.9797
f3, f9	15.522	<0.0001	S4, S8	-0.093	>0.9999
f4, f5	1.184	<0.0074	S4, S9	-0.352	0.9931
f4, f6	8.858	<0.0001	S4, S10	-1.149	0.0171
f4, f7	4.627	<0.0001	S5, S6	-0.026	>0.9999
f4, f8	7.486	<0.0001	S5, S7	0.139	>0.9999
f4, f9	4.934	<0.0001	S5, S8	-0.363	0.9913
f5, f6	7.673	<0.0001	S5, S9	-0.621	0.7509
f5, f7	3.443	<0.0001	S5, S10	-1.419	0.0003
f5, f8	6.301	<0.0001	S6, S7	0.165	>0.9999
f5, f9	3.749	<0.0001	S6, S8	-0.337	0.9951
f6, f7	-4.230	<0.0001	S6, S9	-0.595	0.7980
f6, f8	-1.372	<0.0001	S6, S10	-1.392	0.0005
f6, f9	-3.924	<0.0005	S7, S8	-0.501	0.9217
f7, f8	2.859	<0.0001	S7, S9	-0.760	0.4551
f7, f9	0.307	<0.994	S7, S10	-1.557	<0.0001
f8, f9	-2.552	<0.0001	S8, S9	-0.258	0.9994
Site			S8, S10	-1.056	0.0488
F, C	-0.109	0.7883	S9, S10	-0.797	0.3753
F, P	-0.518	0.0012			
C, P	-0.409	0.0319			

Supplementary Table 16. ANOVA results for SD of the *Strength (S)* across the different guitarists, frequencies, sites, and sequences for Comme un Tango (MP2)

Factors	df	F-value	P-value	η^2
Guitarist	3.900	6.657	0.000	0.022
Frequency	8.900	14567.139	0.000	0.992
Site	2.900	1.966	0.141	0.004
Guitarist * Frequency	24.900	5.125	0.000	0.120
Guitarist * Site	6.900	2.132	0.048	0.014
Frequency * Site	16.900	6.863	0.000	0.109
Guitarist * Frequency * Site	48.900	3.884	0.000	0.172
Error				
Sequence	12.371	7.311	0.000	0.008
Sequence * Guitarist	37.114	6.042	0.000	0.020
Sequence * Frequency	98.970	5.681	0.000	0.048
Sequence * Site	24.742	1.562	0.037	0.003
Sequence * Guitarist * Frequency	296.909	4.581	0.000	0.109
Sequence * Guitarist * Site	74.227	2.624	0.000	0.017
Sequence * Frequency * Site	197.940	2.512	0.000	0.043
Sequence * Guitarist * Frequency * Site	593.819	2.660	0.000	0.124
Error (Sequence)	11134.105			

Supplementary Table 17. Scheffé test for post-hoc differences in SD of the *Strength (S)* between different factor levels for the factors Guitarist, Frequency, Site and Sequence in Comme un tango (MP2)

Levels	M. Diff.	P-Value	Levels	M. Diff.	P-Value	Levels	M. Diff.	P-Value
Guitarist			Sequence					
A, B	0.37	0.0785	S1, S2	0.062	>0.999	S5, S7	1.099	0.1525
A, C	0.605	0.0004	S1, S3	0.959	0.3868	S5, S8	0.567	0.9788
A, D	0.148	0.7780	S1, S4	-0.161	>0.999	S5, S9	0.242	>0.999
B, C	0.235	0.4298	S1, S5	0.015	>0.999	S5, S10	1.011	0.2852
B, D	-0.221	0.4849	S1, S6	0.611	0.9592	S5, S11	0.482	0.9955
C, D	-0.457	0.0157	S1, S7	1.114	0.1350	S5, S12	0.330	>0.999
Frequency			S1, S8	0.582	0.9733	S5, S13	1.560	0.0005
f1, f2	18.684	<0.0001	S1, S9	0.257	>0.999	S5, S14	0.485	0.9952
f1, f3	34.522	<0.0001	S1, S10	1.026	0.2590	S6, S7	0.503	0.9932
f1, f4	46.138	<0.0001	S1, S11	0.497	0.9939	S6, S8	-0.029	>0.999
f1, f5	46.846	<0.0001	S1, S12	0.345	0.9999	S6, S9	-0.354	0.9998
f1, f6	54.121	<0.0001	S1, S13	1.575	0.0004	S6, S10	0.415	0.9991
f1, f7	50.013	<0.0001	S1, S14	0.500	0.9935	S6, S11	-0.114	>0.999
f1, f8	52.493	<0.0001	S2, S3	0.897	0.5198	S6, S12	-0.266	>0.999
f1, f9	50.158	<0.0001	S2, S4	-0.223	>0.999	S6, S13	0.964	0.3776
f2, f3	15.838	<0.0001	S2, S5	-0.047	>0.999	S6, S14	-0.111	>0.999
f2, f4	27.454	<0.0001	S2, S6	0.549	0.9841	S7, S8	-0.532	0.9882
f2, f5	28.163	<0.0001	S2, S7	1.052	0.2172	S7, S9	-0.857	0.6091
f2, f6	35.437	<0.0001	S2, S8	0.520	0.9905	S7, S10	-0.087	>0.999
f2, f7	31.329	<0.0001	S2, S9	0.196	>0.999	S7, S11	-0.617	0.9559
f2, f8	33.809	<0.0001	S2, S10	0.965	0.3757	S7, S12	-0.768	0.7826
f2, f9	31.474	<0.0001	S2, S11	0.435	0.9984	S7, S13	0.461	0.9971
f3, f4	11.616	<0.0001	S2, S12	0.284	>0.999	S7, S14	-0.613	0.9579
f3, f5	12.325	<0.0001	S2, S13	1.513	0.0011	S8, S9	-0.324	>0.999
f3, f6	19.599	<0.0001	S2, S14	0.439	0.9983	S8, S10	0.445	0.9980
f3, f7	15.491	<0.0001	S3, S4	-1.120	0.1282	S8, S11	-0.085	>0.999
f3, f8	17.971	<0.0001	S3, S5	-0.944	0.4181	S8, S12	-0.236	>0.999
f3, f9	15.636	<0.0001	S3, S6	-0.348	0.9999	S8, S13	0.993	0.3192
f4, f5	0.709	0.1948	S3, S7	0.155	>0.999	S8, S14	-0.081	>0.999
f4, f6	7.983	<0.0001	S3, S8	-0.377	0.9997	S9, S10	0.769	0.7816
f4, f7	3.876	<0.0001	S3, S9	-0.702	0.8803	S9, S11	0.240	>0.999
f4, f8	6.355	<0.0001	S3, S10	0.067	>0.999	S9, S12	0.088	>0.999
f4, f9	4.020	<0.0001	S3, S11	-0.462	0.9971	S9, S13	1.317	0.0167
f5, f6	7.274	<0.0001	S3, S12	-0.614	0.9577	S9, S14	0.243	>0.999
f5, f7	3.167	<0.0001	S3, S13	0.616	0.9566	S10, S11	-0.529	0.9888
f5, f8	5.646	<0.0001	S3, S14	-0.459	0.9973	S10, S12	-0.681	0.9039
f5, f9	3.311	<0.0001	S4, S5	0.176	>0.999	S10, S13	0.548	0.9843
f6, f7	-4.108	<0.0001	S4, S6	0.772	0.7764	S10, S14	-0.526	0.9894
f6, f8	-1.628	<0.0001	S4, S7	1.275	0.0277	S11, S12	-0.152	>0.999
f6, f9	-3.963	<0.0001	S4, S8	0.743	0.8246	S11, S13	1.078	0.1800
f7, f8	2.479	<0.0001	S4, S9	0.418	0.9990	S11, S14	0.003	>0.999
f7, f9	0.145	>0.999	S4, S10	1.187	0.0698	S12, S13	1.229	0.0455
f8, f9	-2.335	<0.0001	S4, S11	0.658	0.9258	S12, S14	0.155	>0.999
Site			S4, S12	0.506	0.9927	S13, S14	-1.074	0.1845
F, C	-0.045	<0.9416	S4, S13	1.736	<0.0001			
F, P	-0.219	0.1681	S4, S14	0.661	0.9229			
C, P	-0.173	0.3999	S5, S6	0.596	0.9669			

Supplementary Table 18. ANOVA results for SD of the *Clustering Coefficient (CC)* across the different guitarists, frequencies, sites, and sequences for Libertango (MP1)

Factors	df	F-value	P-value	η^2
Guitarist	3	86.417	0.000	0.224
Frequency	8	2902.261	0.000	0.963
Site	2	8.290	0.000	0.018
Guitarist * Frequency	24	9.426	0.000	0.201
Guitarist * Site	6	6.283	0.000	0.040
Frequency * Site	16	2.106	0.007	0.036
Guitarist * Frequency * Site	48	3.529	0.000	0.158
Error				
Sequence	8.082	161.018	0.000	0.152
Sequence * Guitarist	24.246	8.246	0.000	0.027
Sequence * Frequency	64.656	14.010	0.000	0.111
Sequence * Site	16.164	3.201	0.000	0.007
Sequence * Guitarist * Frequency	193.968	7.934	0.000	0.175
Sequence * Guitarist * Site	48.492	2.501	0.000	0.016
Sequence * Frequency * Site	129.312	2.696	0.000	0.046
Sequence * Guitarist * Frequency * Site	387.936	2.115	0.000	0.101
Error (Sequence)	7273.791			

Supplementary Table 19. Scheffé test for post-hoc differences in SD of the *Clustering Coefficient (CC)* between different factor levels for the factors Guitarist, Frequency, Site and Sequence in Libertango (MP1)

Levels	M. Diff.	P-Value	Levels	M. Diff.	P-Value
Guitarist			Sequence		
A, B	-0.0003	<0.0001	S1, S2	0.00006	0.9929
A, C	-0.0003	<0.0001	S1, S3	0.001	<0.0001
A, D	-0.001	<0.0001	S1, S4	-0.0001	0.1086
B, C	0.00001	0.9759	S1, S5	0.001	<0.0001
B, D	-0.0002	<0.0001	S1, S6	-0.001	<0.0001
C, D	-0.0002	<0.0001	S1, S7	-0.0003	<0.0001
Frequency			S1, S8	9.9E-7	>0.999
f1, f2	0.003	<0.0001	S1, S9	0.00005	0.9970
f1, f3	0.005	<0.0001	S1, S10	0.0002	0.0446
f1, f4	0.005	<0.0001	S2, S3	0.001	<0.0001
f1, f5	0.006	<0.0001	S2, S4	-0.0002	0.0015
f1, f6	0.004	<0.0001	S2, S5	0.0006	<0.0001
f1, f7	0.006	<0.0001	S2, S6	-0.001	<0.0001
f1, f8	0.005	<0.0001	S2, S7	-0.0004	<0.0001
f1, f9	0.007	<0.0001	S2, S8	-0.00006	0.9921
f2, f3	0.002	<0.0001	S2, S9	-0.00001	>0.999
f2, f4	0.002	<0.0001	S2, S10	0.0001	0.5626
f2, f5	0.002	<0.0001	S3, S4	-0.001	<0.0001
f2, f6	0.001	<0.0001	S3, S5	-0.0004	<0.0001
f2, f7	0.003	<0.0001	S3, S6	-0.002	<0.0001
f2, f8	0.002	<0.0001	S3, S7	-0.001	<0.0001
f2, f9	0.003	<0.0001	S3, S8	-0.001	<0.0001
f3, f4	0.0003	<0.0001	S3, S9	-0.001	<0.0001
f3, f5	0.001	<0.0001	S3, S10	-0.001	<0.0001
f3, f6	-0.001	<0.0001	S4, S5	0.001	<0.0001
f3, f7	0.001	<0.0001	S4, S6	-0.0005	<0.0001
f3, f8	0.001	<0.0001	S4, S7	-0.0001	0.6341
f3, f9	0.002	<0.0001	S4, S8	0.0002	0.1138
f4, f5	0.0004	<0.0001	S4, S9	0.0002	0.0027
f4, f6	-0.001	<0.0001	S4, S10	0.0004	<0.0001
f4, f7	0.001	<0.0001	S5, S6	-0.001	<0.0001
f4, f8	0.0003	<0.0001	S5, S7	-0.001	<0.0001
f4, f9	0.001	<0.0001	S5, S8	-0.001	<0.0001
f5, f6	-0.002	<0.0001	S5, S9	-0.0005	<0.0001
f5, f7	0.0003	<0.0001	S5, S10	-0.0003	<0.0001
f5, f8	-0.0001	0.7302	S6, S7	0.0003	<0.0001
f5, f9	0.001	<0.0001	S6, S8	0.001	<0.0001
f6, f7	0.002	<0.0001	S6, S9	0.001	<0.0001
f6, f8	0.001	<0.0001	S6, S10	0.001	<0.0001
f6, f9	0.002	<0.0001	S7, S8	0.0003	<0.0001
f7, f8	-0.0004	<0.0001	S7, S9	0.0004	<0.0001
f7, f9	0.001	<0.0001	S7, S10	0.0005	<0.0001
f8, f9	0.001	<0.0001	S8, S9	0.00006	0.9966
Site			S8, S10	0.0002	0.0422
F, C	-0.00008	0.0343	S9, S10	0.0002	0.4782
F, P	-0.0001	0.0004			
C, P	-0.00003	0.6387			

Supplementary Table 20. ANOVA results for SD of the *Clustering Coefficient (CC)* across the different guitarists, frequencies, sites, and sequences for Comme un Tango (MP2)

Factors	df	F-value	P-value	η^2
Guitarist	3	64.801	0.000	0.178
Frequency	8	3285.287	0.000	0.967
Site	2	4.021	0.018	0.009
Guitarist * Frequency	24	10.000	0.000	0.211
Guitarist * Site	6	5.627	0.000	0.036
Frequency * Site	16	5.014	0.000	0.082
Guitarist * Frequency * Site	48	2.764	0.000	0.128
Error	900			
Sequence	12.535	242.688	0.000	0.212
Sequence * Guitarist	37.606	15.811	0.000	0.050
Sequence * Frequency	100.283	19.528	0.000	0.148
Sequence * Site	25.071	3.145	0.000	0.007
Sequence * Guitarist * Frequency	300.849	8.924	0.000	0.192
Sequence * Guitarist * Site	75.212	2.369	0.000	0.016
Sequence * Frequency * Site	200.566	3.188	0.000	0.054
Sequence * Guitarist * Frequency * Site	601.697	2.459	0.000	0.116
Error (Sequence)	11281.819			

Supplementary Table 21. Scheffé test for post-hoc differences in SD of the *Clustering Coefficient (CC)* between different factor levels for the factors Guitarist, Frequency, Site and Sequence in Comme un tango (MP2)

Levels	M. Diff.	P-Value	Levels	M. Diff.	P-Value	Levels	M. Diff.	P-Value
Guitarist			Sequence					
A, B	0.00007	0.1471	S1, S2	-0.001	<0.0001	S5, S7	0.002	<0.0001
A, C	0.0003	<0.0001	S1, S3	0.0002	0.2602	S5, S8	0.001	<0.0001
A, D	-0.00008	0.0704	S1, S4	-0.001	<0.0001	S5, S9	0.001	<0.0001
B, C	0.0002	<0.0001	S1, S5	-0.002	<0.0001	S5, S10	0.002	<0.0001
B, D	-0.0001	<0.0001	S1, S6	-0.00005	>0.999	S5, S11	0.001	<0.0001
C, D	-0.0004	<0.0001	S1, S7	0.0004	<0.0001	S5, S12	0.001	<0.0001
Frequency			S1, S8	-0.001	<0.0001	S5, S13	0.002	<0.0001
f1, f2	0.002	<0.0001	S1, S9	-0.0004	<0.0001	S5, S14	0.001	<0.0001
f1, f3	0.004	<0.0001	S1, S10	0.0002	0.0865	S6, S7	0.0004	<0.0001
f1, f4	0.004	<0.0001	S1, S11	-0.00002	>0.999	S6, S8	-0.001	<0.0001
f1, f5	0.005	<0.0001	S1, S12	-0.00004	>0.999	S6, S9	-0.0004	<0.0001
f1, f6	0.003	<0.0001	S1, S13	0.001	<0.0001	S6, S10	0.0003	0.0034
f1, f7	0.005	<0.0001	S1, S14	-0.00003	>0.999	S6, S11	0.00003	>0.999
f1, f8	0.005	<0.0001	S2, S3	0.001	<0.0001	S6, S12	0.00001	>0.999
f1, f9	0.006	<0.0001	S2, S4	-0.00002	>0.999	S6, S13	0.001	<0.0001
f2, f3	0.002	<0.0001	S2, S5	-0.001	<0.0001	S6, S14	0.00002	>0.999
f2, f4	0.002	<0.0001	S2, S6	0.001	<0.0001	S7, S8	-0.001	<0.0001
f2, f5	0.003	<0.0001	S2, S7	0.001	<0.0001	S7, S9	-0.001	<0.0001
f2, f6	0.001	<0.0001	S2, S8	-0.00001	>0.999	S7, S10	-0.0001	0.8406
f2, f7	0.003	<0.0001	S2, S9	0.0002	0.3602	S7, S11	-0.0004	<0.0001
f2, f8	0.003	<0.0001	S2, S10	0.001	<0.0001	S7, S12	-0.0004	<0.0001
f2, f9	0.004	<0.0001	S2, S11	0.001	<0.0001	S7, S13	0.0003	<0.0001
f3, f4	0.001	<0.0001	S2, S12	0.001	<0.0001	S7, S14	-0.0004	<0.0001
f3, f5	0.001	<0.0001	S2, S13	0.001	<0.0001	S8, S9	0.0002	0.2983
f3, f6	-0.0002	0.0001	S2, S14	0.001	<0.0001	S8, S10	0.001	<0.0001
f3, f7	0.001	<0.0001	S3, S4	-0.001	<0.0001	S8, S11	0.001	<0.0001
f3, f8	0.001	<0.0001	S3, S5	-0.002	<0.0001	S8, S12	0.001	<0.0001
f3, f9	0.002	<0.0001	S3, S6	-0.0002	0.0211	S8, S13	0.001	<0.0001
f4, f5	0.0005	<0.0001	S3, S7	0.0002	0.5783	S8, S14	0.001	<0.0001
f4, f6	-0.001	<0.0001	S3, S8	-0.001	<0.0001	S9, S10	0.001	<0.0001
f4, f7	0.001	<0.0001	S3, S9	-0.001	<0.0001	S9, S11	0.0004	<0.0001
f4, f8	0.0004	<0.0001	S3, S10	0.00003	>0.999	S9, S12	0.0004	<0.0001
f4, f9	0.002	<0.0001	S3, S11	-0.0002	0.1263	S9, S13	0.001	<0.0001
f5, f6	-0.001	<0.0001	S3, S12	-0.0002	0.0434	S9, S14	0.0004	<0.0001
f5, f7	0.0003	<0.0001	S3, S13	0.0005	<0.0001	S10, S11	-0.0002	0.0324
f5, f8	-0.00002	>0.9999	S3, S14	-0.0002	0.0865	S10, S12	-0.0003	0.0082
f5, f9	0.001	<0.0001	S4, S5	-0.001	<0.0001	S10, S13	0.0005	<0.0001
f6, f7	0.002	<0.0001	S4, S6	0.001	<0.0001	S10, S14	-0.0002	0.0198
f6, f8	0.001	<0.0001	S4, S7	0.001	<0.0001	S11, S12	-0.00002	>0.999
f6, f9	0.003	<0.0001	S4, S8	0.00001	>0.999	S11, S13	0.001	<0.0001
f7, f8	-0.0003	<0.0001	S4, S9	0.0002	0.1854	S11, S14	-0.00001	>0.999
f7, f9	0.001	<0.0001	S4, S10	0.001	<0.0001	S12, S13	0.001	<0.0001
f8, f9	0.001	<0.0001	S4, S11	0.001	<0.0001	S12, S14	0.00001	>0.999
Site			S4, S12	0.001	<0.0001	S13, S14	-0.001	<0.0001
F, C	-0.00005	0.4624	S4, S13	0.001	<0.0001			
F, P	-0.00007	0.0183	S4, S14	0.001	<0.0001			
C, P	-0.00003	0.4325	S5, S6	0.001	<0.0001			

Supplementary Table 22. ANOVA results for SD of the *Characteristic Path Length (CPL)* across the different guitarists, frequencies, sites, and sequences for Libertango (MP1)

Factors	df	F-value	P-value	η^2
Guitarist	3	10.194	0.000	0.033
Frequency	8	3144.820	0.000	0.965
Site	2	1.771	0.171	0.004
Guitarist * Frequency	24	10.862	0.000	0.225
Guitarist * Site	6	1.957	0.069	0.013
Frequency * Site	16	2.706	0.000	0.046
Guitarist * Frequency * Site	48	1.481	0.020	0.073
Error				
Sequence	7.770	11.437	0.000	0.013
Sequence * Guitarist	23.310	5.495	0.000	0.018
Sequence * Frequency	62.161	3.952	0.000	0.034
Sequence * Site	15.540	2.788	0.000	0.006
Sequence * Guitarist * Frequency	186.482	5.289	0.000	0.124
Sequence * Guitarist * Site	46.621	2.120	0.000	0.014
Sequence * Frequency * Site	124.322	2.145	0.000	0.037
Sequence * Guitarist * Frequency * Site	372.965	1.796	0.000	0.087
Error (Sequence)	6993.086			

Supplementary Table 23. Scheffé test for post-hoc differences in SD of the *Characteristic Path Length (CPL)* between different factor levels for the factors Guitarist, Frequency, Site and Sequence in Libertango (MP1)

Levels	M. Diff.	P-Value	Levels	M. Diff.	P-Value
Guitarist			Sequence		
A, B	-0.003	0.0608	S1, S2	0.006	0.1573
A, C	-0.007	<0.0001	S1, S3	0.005	0.6210
A, D	-0.001	0.8379	S1, S4	-0.001	>0.9999
B, C	-0.003	0.1132	S1, S5	0.003	0.9514
B, D	0.002	0.3567	S1, S6	-0.006	0.3640
C, D	0.005	0.0005	S1, S7	-0.005	0.5173
Frequency			S1, S8	0.001	>0.9999
f1, f2	0.162	<0.0001	S1, S9	-0.001	>0.9999
f1, f3	0.210	<0.0001	S1, S10	-0.003	0.9895
f1, f4	0.221	<0.0001	S2, S3	-0.002	0.9996
f1, f5	0.230	<0.0001	S2, S4	-0.007	0.0591
f1, f6	0.224	<0.0001	S2, S5	-0.003	0.9527
f1, f7	0.241	<0.0001	S2, S6	-0.012	<0.0001
f1, f8	0.240	<0.0001	S2, S7	-0.011	<0.0001
f1, f9	0.239	<0.0001	S2, S8	-0.006	0.3303
f2, f3	0.048	<0.0001	S2, S9	-0.007	0.0583
f2, f4	0.059	<0.0001	S2, S10	-0.009	0.0023
f2, f5	0.068	<0.0001	S3, S4	-0.005	0.3819
f2, f6	0.062	<0.0001	S3, S5	-0.002	0.9998
f2, f7	0.079	<0.0001	S3, S6	-0.010	0.0001
f2, f8	0.078	<0.0001	S3, S7	-0.010	0.0004
f2, f9	0.077	<0.0001	S3, S8	-0.004	0.8268
f3, f4	0.011	<0.0001	S3, S9	-0.006	0.3789
f3, f5	0.020	<0.0001	S3, S10	-0.007	0.0481
f3, f6	0.014	<0.0001	S4, S5	0.004	0.8317
f3, f7	0.031	<0.0001	S4, S6	-0.005	0.6019
f3, f8	0.030	<0.0001	S4, S7	-0.004	0.7490
f3, f9	0.029	<0.0001	S4, S8	0.002	0.9999
f4, f5	0.009	0.0063	S4, S9	-0.00001	>0.9999
f4, f6	0.003	0.9722	S4, S10	-0.002	0.9993
f4, f7	0.019	<0.0001	S5, S6	-0.009	0.0036
f4, f8	0.019	<0.0001	S5, S7	-0.008	0.0095
f4, f9	0.018	<0.0001	S5, S8	-0.002	0.9923
f5, f6	-0.006	0.2759	S5, S9	-0.004	0.8295
f5, f7	0.011	0.0002	S5, S10	-0.006	0.2979
f5, f8	0.010	0.0011	S6, S7	0.0005	>0.9999
f5, f9	0.009	0.0059	S6, S8	0.006	0.1794
f6, f7	0.017	<0.0001	S6, S9	0.005	0.6050
f6, f8	0.016	<0.0001	S6, S10	0.003	0.9706
f6, f9	0.015	<0.0001	S7, S8	0.006	0.2937
f7, f8	-0.001	>0.9999	S7, S9	0.004	0.7517
f7, f9	-0.002	0.9993	S7, S10	0.002	0.9919
f8, f9	-0.001	>0.9999	S8, S9	-0.002	0.9998
Site			S8, S10	-0.003	0.9405
F, C	0.0003	0.9523	S9, S10	-0.002	0.9993
F, P	-0.02	0.3261			
C, P	-0.02	0.2474			

Supplementary Table 24. ANOVA results for SD of the *Characteristic Path Length (CPL)* across the different guitarists, frequencies, sites, and sequences for Comme un Tango (MP2)

Factors	df	F-value	P-value	η^2
Guitarist	3	13.671	0.000	0.044
Frequency	8	5165.759	0.000	0.979
Site	2	0.194	0.824	0.000
Guitarist * Frequency	24	12.930	0.000	0.256
Guitarist * Site	6	2.832	0.010	0.019
Frequency * Site	16	6.580	0.000	0.105
Guitarist * Frequency * Site	48	2.193	0.000	0.105
Error	900			
Sequence	12.024	15.260	0.000	0.017
Sequence * Guitarist	36.073	5.865	0.000	0.019
Sequence * Frequency	96.195	6.081	0.000	0.051
Sequence * Site	24.049	2.756	0.000	0.006
Sequence * Guitarist * Frequency	288.586	5.501	0.000	0.128
Sequence * Guitarist * Site	72.147	2.755	0.000	0.018
Sequence * Frequency * Site	192.391	3.175	0.000	0.053
Sequence * Guitarist * Frequency * Site	577.173	2.734	0.000	0.127
Error (Sequence)	10821.984			

Supplementary Table 25. Scheffé test for post-hoc differences in SD of the *Characteristic Path Length (CPL)* between different factor levels for the factors Guitarist, Frequency, Site and Sequence in Comme un tango (MP2)

Levels	M. Diff.	P-Value	Levels	M. Diff.	P-Value	Levels	M. Diff.	P-Value
Guitarist			Sequence					
A, B	-0.0004	0.9829	S1, S2	-0.003	0.9886	S5, S7	0.011	<0.0001
A, C	0.005	<0.0001	S1, S3	0.003	0.9956	S5, S8	0.008	0.0243
A, D	0.002	0.1246	S1, S4	-0.003	0.9909	S5, S9	0.002	>0.9999
B, C	0.006	<0.0001	S1, S5	-0.012	<0.0001	S5, S10	0.011	<0.0001
B, D	0.003	0.0492	S1, S6	-0.002	0.9998	S5, S11	0.00006	>0.9999
C, D	-0.003	0.0115	S1, S7	-0.001	>0.9999	S5, S12	0.006	0.3816
Frequency			S1, S8	-0.003	0.9848	S5, S13	0.014	<0.0001
f1, f2	0.133	<0.0001	S1, S9	-0.010	0.0008	S5, S14	0.007	0.1743
f1, f3	0.185	<0.0001	S1, S10	-0.001	>0.9999	S6, S7	0.001	>0.9999
f1, f4	0.202	<0.0001	S1, S11	-0.012	<0.0001	S6, S8	-0.001	>0.9999
f1, f5	0.211	<0.0001	S1, S12	-0.006	0.5776	S6, S9	-0.007	0.0809
f1, f6	0.200	<0.0001	S1, S13	0.002	>0.9999	S6, S10	0.001	>0.9999
f1, f7	0.218	<0.0001	S1, S14	-0.005	0.8117	S6, S11	-0.009	0.0023
f1, f8	0.217	<0.0001	S2, S3	0.006	0.2855	S6, S12	-0.003	0.9918
f1, f9	0.216	<0.0001	S2, S4	0.00008	>0.9999	S6, S13	0.004	0.9285
f2, f3	0.052	<0.0001	S2, S5	-0.008	0.0200	S6, S14	-0.003	0.9995
f2, f4	0.069	<0.0001	S2, S6	0.001	>0.9999	S7, S8	-0.003	0.9994
f2, f5	0.078	<0.0001	S2, S7	0.002	0.9996	S7, S9	-0.009	0.0070
f2, f6	0.067	<0.0001	S2, S8	-0.0001	>0.9999	S7, S10	0.00001	>0.9999
f2, f7	0.085	<0.0001	S2, S9	-0.006	0.3049	S7, S11	-0.011	<0.0001
f2, f8	0.083	<0.0001	S2, S10	0.002	0.9996	S7, S12	-0.005	0.8504
f2, f9	0.083	<0.0001	S2, S11	-0.008	0.0225	S7, S13	0.003	0.9982
f3, f4	0.018	<0.0001	S2, S12	-0.002	0.9999	S7, S14	-0.004	0.9626
f3, f5	0.026	<0.0001	S2, S13	0.005	0.6735	S8, S9	-0.006	0.3367
f3, f6	0.015	<0.0001	S2, S14	-0.001	>0.9999	S8, S10	0.003	0.9993
f3, f7	0.033	<0.0001	S3, S4	-0.006	0.3078	S8, S11	-0.008	0.0272
f3, f8	0.032	<0.0001	S3, S5	-0.015	<0.0001	S8, S12	-0.002	>0.9999
f3, f9	0.031	<0.0001	S3, S6	-0.005	0.6438	S8, S13	0.005	0.6389
f4, f5	0.009	<0.0001	S3, S7	-0.004	0.9492	S8, S14	-0.001	>0.9999
f4, f6	-0.002	0.9156	S3, S8	-0.007	0.2563	S9, S10	0.009	0.0069
f4, f7	0.016	<0.0001	S3, S9	-0.013	<0.0001	S9, S11	-0.002	>0.9999
f4, f8	0.014	<0.0001	S3, S10	-0.004	0.9500	S9, S12	0.004	0.9244
f4, f9	0.014	<0.0001	S3, S11	-0.015	<0.0001	S9, S13	0.012	<0.0001
f5, f6	-0.011	<0.0001	S3, S12	-0.009	0.0108	S9, S14	0.005	0.7627
f5, f7	0.007	0.0013	S3, S13	-0.001	>0.9999	S10, S11	-0.011	<0.0001
f5, f8	0.006	0.0396	S3, S14	-0.008	0.0442	S10, S12	-0.005	0.8487
f5, f9	0.005	0.0818	S4, S5	-0.008	0.0172	S10, S13	0.003	0.9982
f6, f7	0.018	<0.0001	S4, S6	0.001	>0.9999	S10, S14	-0.004	0.9620
f6, f8	0.017	<0.0001	S4, S7	0.002	0.9997	S11, S12	0.006	0.4023
f6, f9	0.016	<0.0001	S4, S8	-0.0002	>0.9999	S11, S13	0.014	<0.0001
f7, f8	-0.001	0.9977	S4, S9	-0.006	0.2827	S11, S14	0.007	0.1879
f7, f9	-0.002	0.9878	S4, S10	0.002	0.9997	S12, S13	0.007	0.0845
f8, f9	-0.0004	>0.9999	S4, S11	-0.008	0.0194	S12, S14	0.001	>0.9999
Site			S4, S12	-0.002	0.9998	S13, S14	-0.007	0.2253
F, C	0.0005	0.8471	S4, S13	0.005	0.6981			
F, P	0.00004	0.9983	S4, S14	-0.001	>0.9999			
C, P	-0.0004	0.8666	S5, S6	0.009	0.0020			

Supplementary Table 26. ANOVA results for the *WFC Strength* across the different guitarists, frequencies, sites, coupling types (within vs. between brains) and music pieces (MP1 vs. MP2)

Factors	df	F-value	P-value	η^2
Guitarist	3,900	388.191	<0.0001	0.564
Frequency	8,900	367.326	<0.0001	0.766
Site	2,900	12.694	<0.0001	0.027
Guitarist * Frequency	24,900	5.763	<0.0001	0.133
Guitarist * Site	6,900	11.308	<0.0001	0.070
Frequency * Site	16,900	6.685	<0.0001	0.106
Guitarist * Frequency * Site	48,900	1.640	<0.0001	0.080
Error	900			
Coupling	1	20567.847	<0.0001	0.958
Coupling * Guitarist	3	366.743	<0.0001	0.550
Coupling * Frequency	8	199.804	<0.0001	0.640
Coupling * Site	2	11.668	<0.0001	0.025
Coupling * Guitarist * Frequency	24	5.255	<0.0001	0.123
Coupling * Guitarist * Site	6	10.590	<0.0001	0.066
Coupling * Frequency * Site	16	6.235	<0.0001	0.100
Coupling * Guitarist * Frequency * Site	48	1.659	0.0038	0.081
MP	1	780.022	<0.0001	0.464
MP * Guitarist	3	244.227	<0.0001	0.449
MP * Frequency	8	25.740	<0.0001	0.186
MP * Site	2	39.400	<0.0001	0.081
MP * Guitarist * Frequency	24	17.636	<0.0001	0.320
MP * Guitarist * Site	6	28.693	<0.0001	0.161
MP * Frequency * Site	16	1.963	0.0130	0.034
MP * Guitarist * Frequency * Site	48	0.909	0.6501	0.046
Coupling * MP	1	522.513	<0.0001	0.367
Coupling * MP * Guitarist	3	214.133	<0.0001	0.416
Coupling * MP * Frequency	8	26.476	<0.0001	0.191
Coupling * MP * Site	2	53.615	<0.0001	0.106
Coupling * MP * Guitarist * Frequency	24	19.981	<0.0001	0.348
Coupling * MP * Guitarist * Site	6	31.616	<0.0001	0.174
Coupling * MP * Frequency * Site	16	1.034	0.4169	0.018
Coupling * MP * Guitarist * Frequency * Site	48	0.995	0.4839	0.050
Error (Coupling * MP)	900			

MP, Music Piece

Supplementary Table 27. ANOVA results for the *CFC Strength* across the different guitarists, frequencies, sites, coupling types (within vs. between brains) and music pieces (MP1 vs. MP2)

Factors	df	F-value	P-value	η^2
Guitarist	3,900	209.830	<0.0001	0.412
Frequency	8,900	22410.455	<0.0001	0.995
Site	2,900	3.727	0.024	0.008
Guitarist * Frequency	24,900	17.718	<0.0001	0.321
Guitarist * Site	6,900	3.238	0.004	0.021
Frequency * Site	16,900	15.898	<0.0001	0.220
Guitarist * Frequency * Site	48,900	4.649	<0.0001	0.199
Error	900			
Coupling	1	124.622	<0.0001	0.122
Coupling * Guitarist	3	339.224	<0.0001	0.531
Coupling * Frequency	8	11.854	<0.0001	0.095
Coupling * Site	2	3.674	0.026	0.008
Coupling * Guitarist * Frequency	24	26.033	<0.0001	0.410
Coupling * Guitarist * Site	6	5.915	<0.0001	0.038
Coupling * Frequency * Site	16	1.516	0.087	0.026
Coupling * Guitarist * Frequency * Site	48	7.013	<0.0001	0.272
MP	1	643.248	<0.0001	0.417
MP * Guitarist	3	78.731	<0.0001	0.208
MP * Frequency	8	125.987	<0.0001	0.528
MP * Site	2	2.654	0.071	0.006
MP * Guitarist * Frequency	24	10.830	<0.0001	0.224
MP * Guitarist * Site	6	2.881	0.009	0.019
MP * Frequency * Site	16	12.068	<0.0001	0.177
MP * Guitarist * Frequency * Site	48	5.057	<0.0001	0.212
Coupling * MP	1	19.486	<0.0001	0.021
Coupling * MP * Guitarist	3	62.823	<0.0001	0.173
Coupling * MP * Frequency	8	1.423	0.183	0.012
Coupling * MP * Site	2	1.702	0.183	0.004
Coupling * MP * Guitarist * Frequency	24	11.348	<0.0001	0.232
Coupling * MP * Guitarist * Site	6	2.339	0.030	0.015
Coupling * MP * Frequency * Site	16	1.444	0.114	0.025
Coupling * MP * Guitarist * Frequency * Site	48	5.087	<0.0001	0.213
Error (Coupling * MP)	900			

MP, Music Piece

Supplementary Table 28. Scheffé test for post-hoc differences in *WFC* and *CFC Strength* between different factor levels for the factors Guitarist, Frequency, and Site.

	WFC		CFC	
Levels	M. Diff.	P-Value	M. Diff.	P-Value
Guitarist				
A, B	-1.298	<0.0001	1.382	<0.0001
A, C	-0.811	<0.0001	0.415	<0.0001
A, D	-2.530	<0.0001	0.332	<0.0001
B, C	0.487	<0.0001	-0.967	<0.0001
B, D	-1.233	<0.0001	-1.050	<0.0001
C, D	-1.719	<0.0001	-0.083	0.5612
Frequency				
f1, f2	-2.870	<0.0001	-6.558	<0.0001
f1, f3	-4.593	<0.0001	-2.617	<0.0001
f1, f4	-4.605	<0.0001	8.048	<0.0001
f1, f5	-4.409	<0.0001	5.672	<0.0001
f1, f6	-4.397	<0.0001	22.036	<0.0001
f1, f7	-4.299	<0.0001	10.088	<0.0001
f1, f8	-5.187	<0.0001	16.655	<0.0001
f1, f9	-4.971	<0.0001	14.130	<0.0001
f2, f3	-1.722	<0.0001	3.941	<0.0001
f2, f4	-1.735	<0.0001	14.605	<0.0001
f2, f5	-1.539	<0.0001	12.229	<0.0001
f2, f6	-1.527	<0.0001	28.594	<0.0001
f2, f7	-1.429	<0.0001	16.645	<0.0001
f2, f8	-2.317	<0.0001	23.213	<0.0001
f2, f9	-2.101	<0.0001	20.688	<0.0001
f3, f4	-0.012	>0.999	10.665	<0.0001
f3, f5	0.184	0.9616	8.289	<0.0001
f3, f6	0.196	0.9440	24.653	<0.0001
f3, f7	0.293	0.6061	12.705	<0.0001
f3, f8	-0.594	0.0011	19.272	<0.0001
f3, f9	-0.378	0.2274	16.747	<0.0001
f4, f5	0.196	0.9437	-2.376	<0.0001
f4, f6	0.208	0.9210	13.989	<0.0001
f4, f7	0.306	0.5470	2.040	<0.0001
f4, f8	-0.582	0.0017	8.607	<0.0001
f4, f9	-0.366	0.2719	6.083	<0.0001
f5, f6	0.012	>0.999	16.365	<0.0001
f5, f7	0.110	0.9988	4.416	<0.0001
f5, f8	-0.778	<0.0001	10.983	<0.0001
f5, f9	-0.562	0.0032	8.459	<0.0001
f6, f7	0.098	>0.999	-11.949	<0.0001
f6, f8	-0.790	<0.0001	-5.381	<0.0001
f6, f9	-0.574	0.0022	-7.906	<0.0001
f7, f8	-0.888	<0.0001	6.567	<0.0001
f7, f9	-0.672	<0.0001	4.043	<0.0001
f8, f9	0.216	0.9024	-2.525	<0.0001
Site				
F, C	-0.349	<0.0001	-0.063	0.499
F, P	-0.214	0.0035	0.077	0.2639
C, P	0.135	0.1564	0.141	0.0281

Supplementary Figures

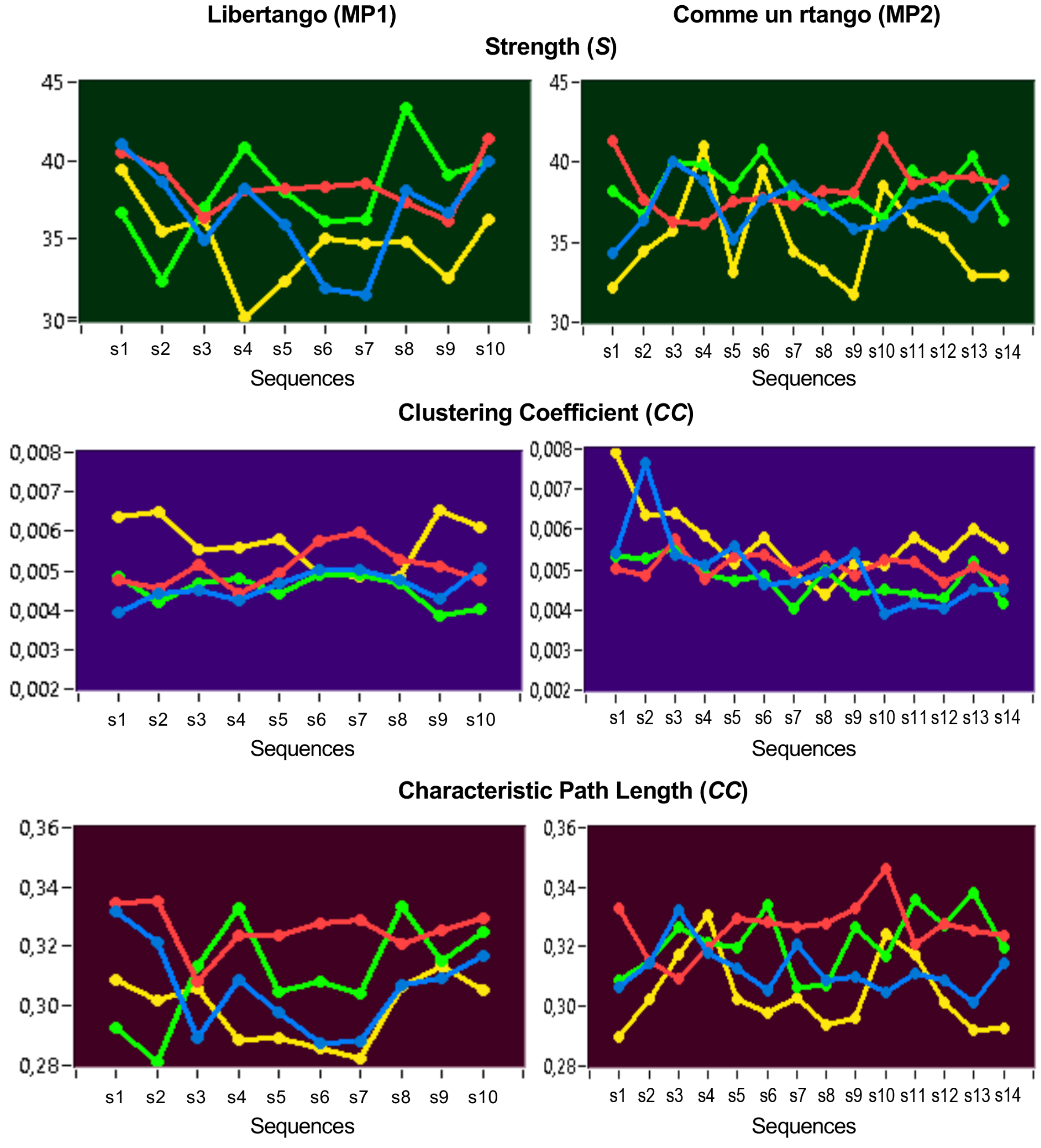

Supplementary Figure 1. Changes in the standard deviation (SD) of the network topology dynamics indices. The SD for the three GTA measures (*S*, *CC*, and *CPL*), averaged separately for the four guitarists' brains (guitarist A in blue, guitarist B in red, guitarist C in green, and guitarist D in yellow), is depicted across the 10 music sequences for Libertango and across the 14 music sequences for Comme un tango, respectively.

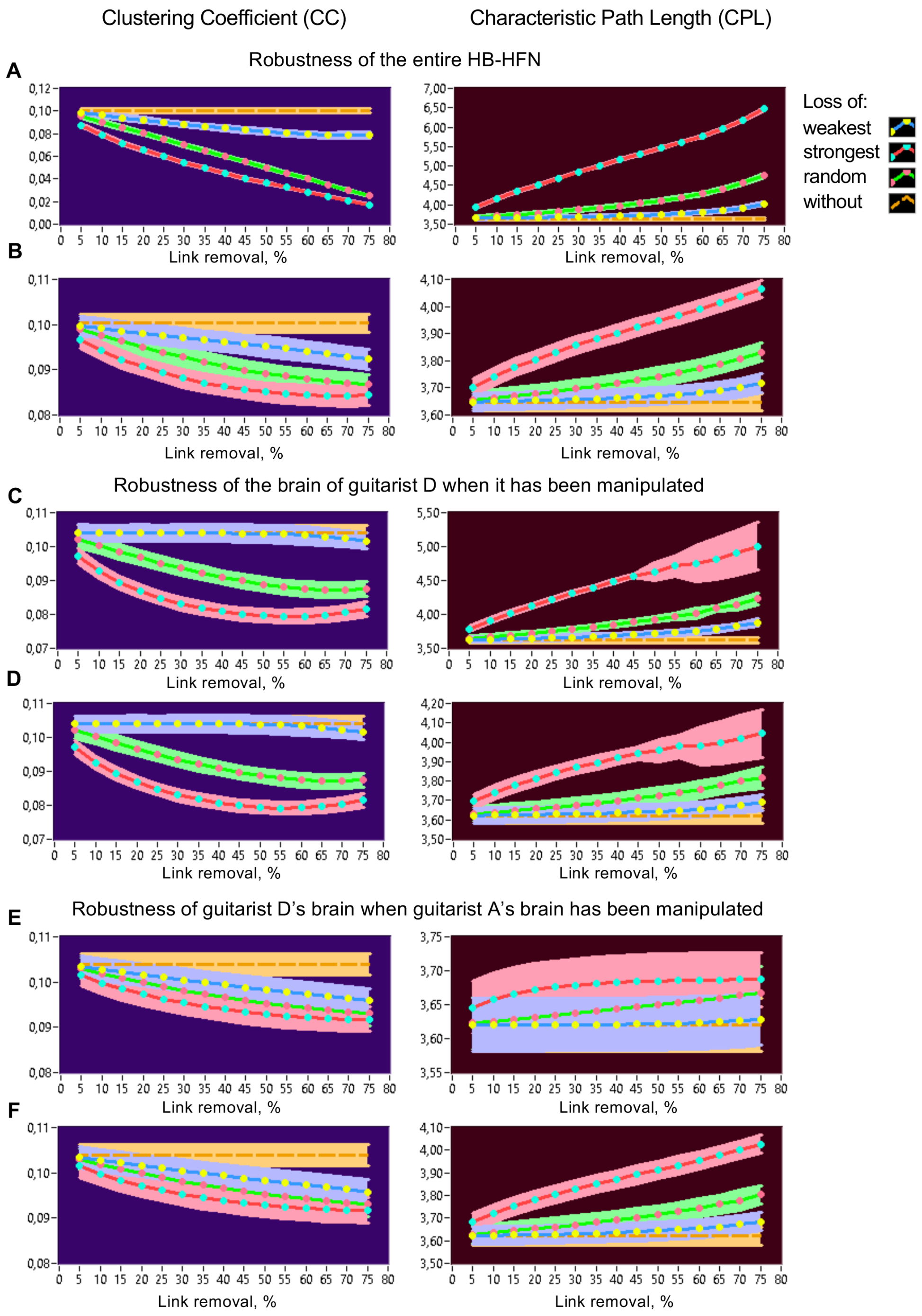

Supplementary Figure 2. Robustness of the entire HB-HFN and of an individual guitarist's brain indicated by changes in *CC* and *CPL* as a function of link removal of different types. (A) Robustness of the entire HB-HFN when the entire HB-HFN has been manipulated. (B) Robustness of the entire HB-HFN when links have been removed only in one guitarist's brain (here guitarist D). (C) Robustness of one guitarist's brain (here guitarist D) as a part of the HB-HFN when in-degree in the same guitarist has been manipulated. (D) Robustness of the one guitarist's brain (here guitarist D) as a part of the HB-HFN when out-degree in the same guitarist has been manipulated. (E) Robustness of one guitarist's brain (here guitarist D) as a part of the HB-HFN when in-degree in another guitarist (here guitarist A) has been manipulated. (F) Robustness of the one guitarist's brain (here guitarist D) as a part of the HB-HFN when out-degree in another guitarist (here guitarist A) has been manipulated. Changes in *CC* (left) and *CPL* (right) as a function of link removal across the 15 5%-steps are presented in all diagrams for different types of link removal: loss of weakest, strongest, and random connections, in comparison to without removal.
